# Supplementary material for: Comparative mitogenomic analysis of subterranean and surface amphipods (Crustacea, Amphipoda) with special reference to the family Crangonyctidae
Source: BMC Genomics. 2024 Mar 20;25:298. doi: 10.1186/s12864-024-10111-w (PMC10956265; doi:10.1186/s12864-024-10111-w)
Supplement: Supplementary file 1 — Supplementary Material 1. [file 12864_2024_10111_MOESM1_ESM.docx]

**Comparative mitogenomic analysis of subterranean and surface amphipods (Crustacea, Amphipoda) with special reference to the family Crangonyctidae**

**Supplementary Materials**


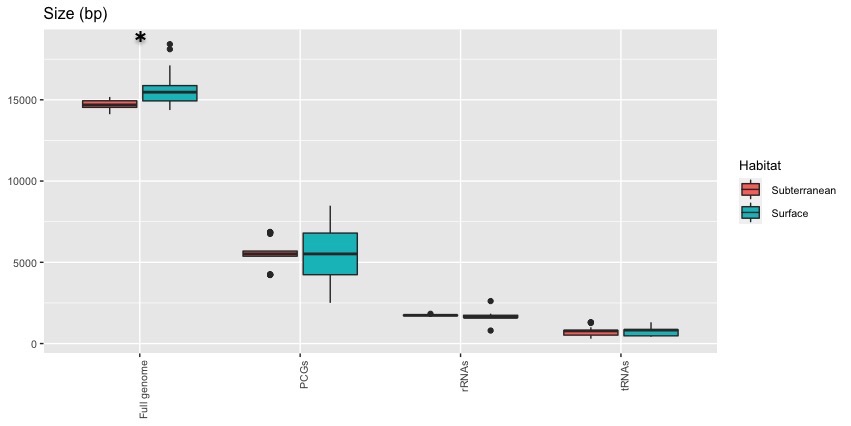


**Supplementary Figure S1.** Box plot showing size (i.e., length) in bp of mitogenomes, protein coding genes (PCG), ribosomal (rRNAs) loci, and transfer ribosomal (tRNAs) loci between subterranean and surface amphipods. Significant P-value < 0.05 is indicated using *.

*
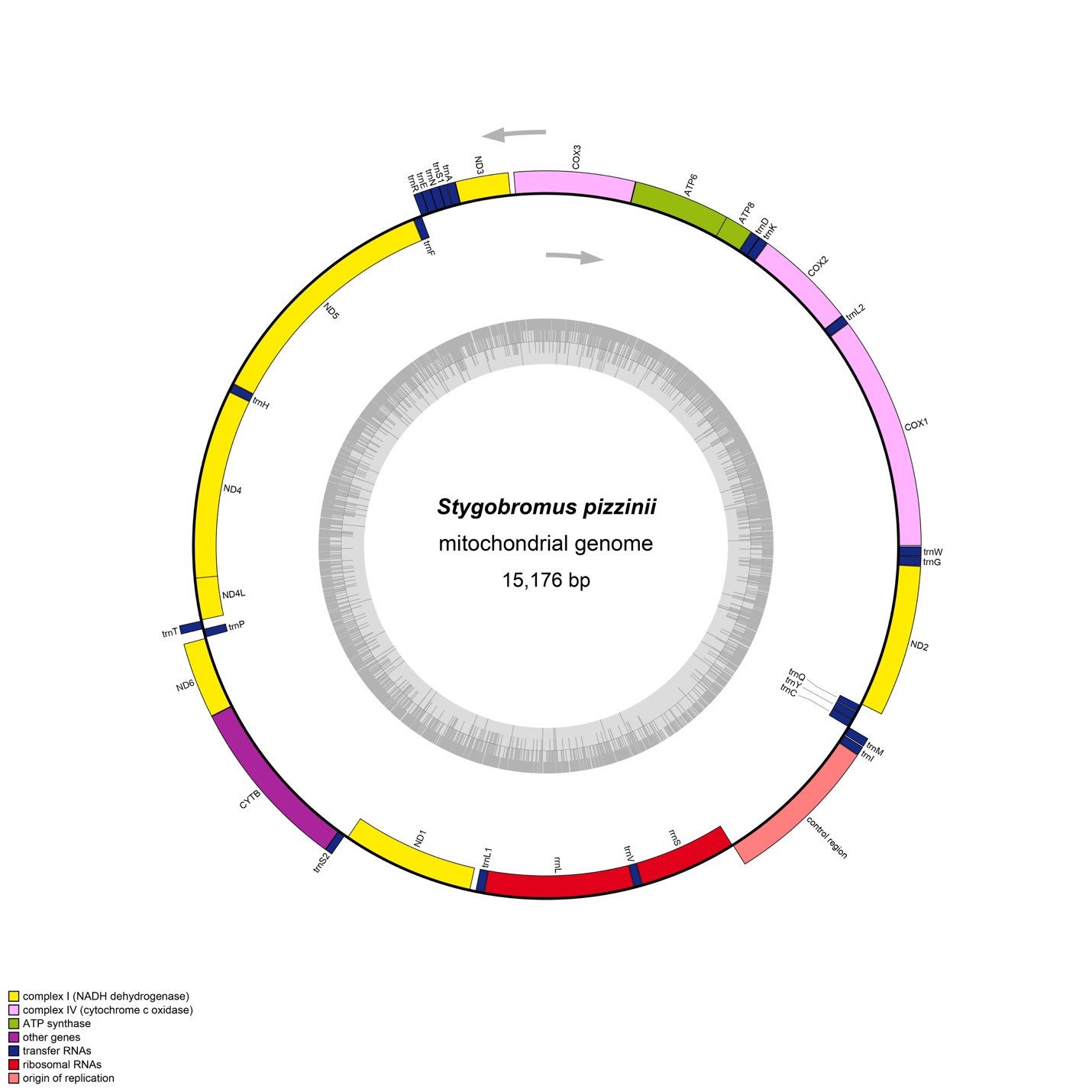
*

*
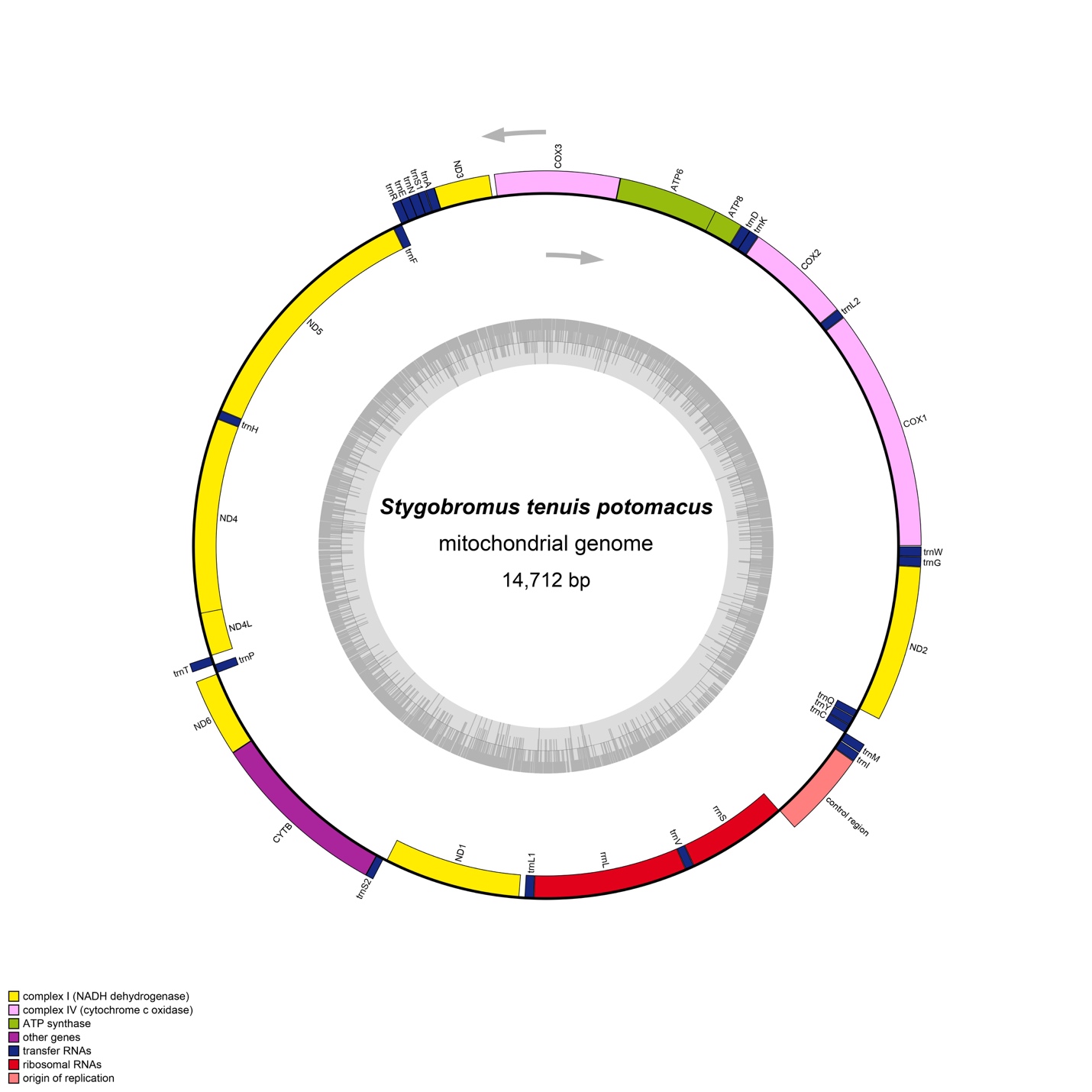
*

*
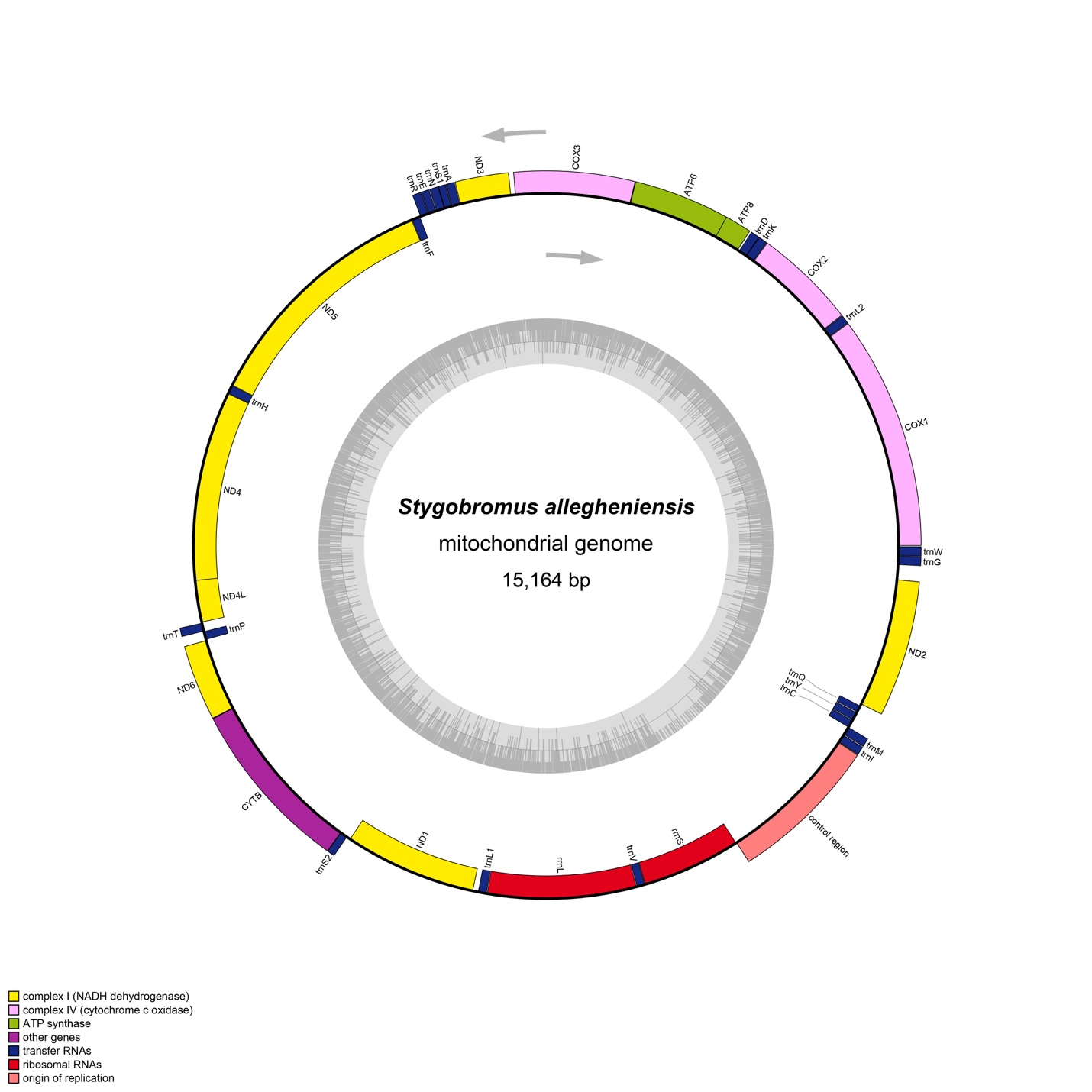
*

*
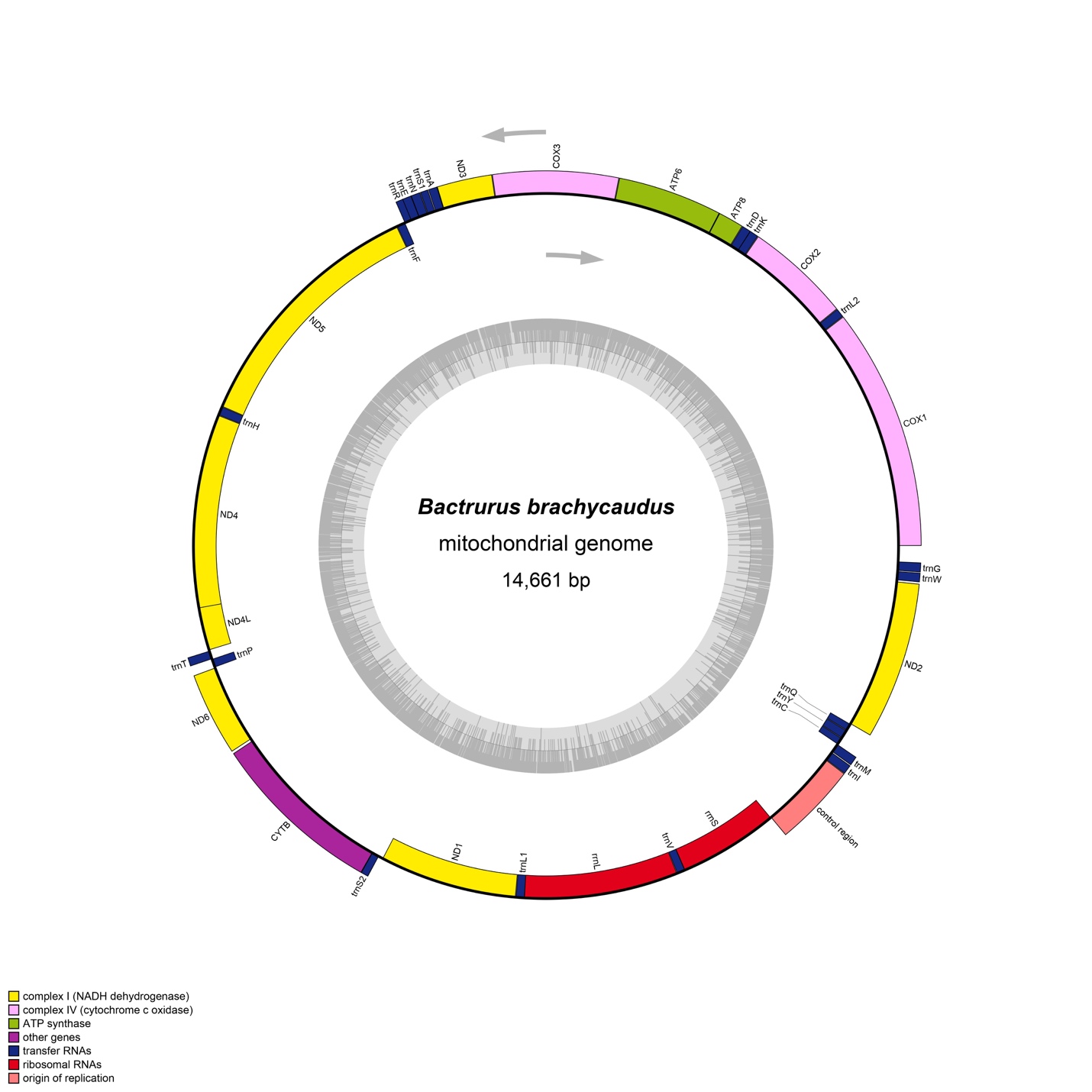
*

*
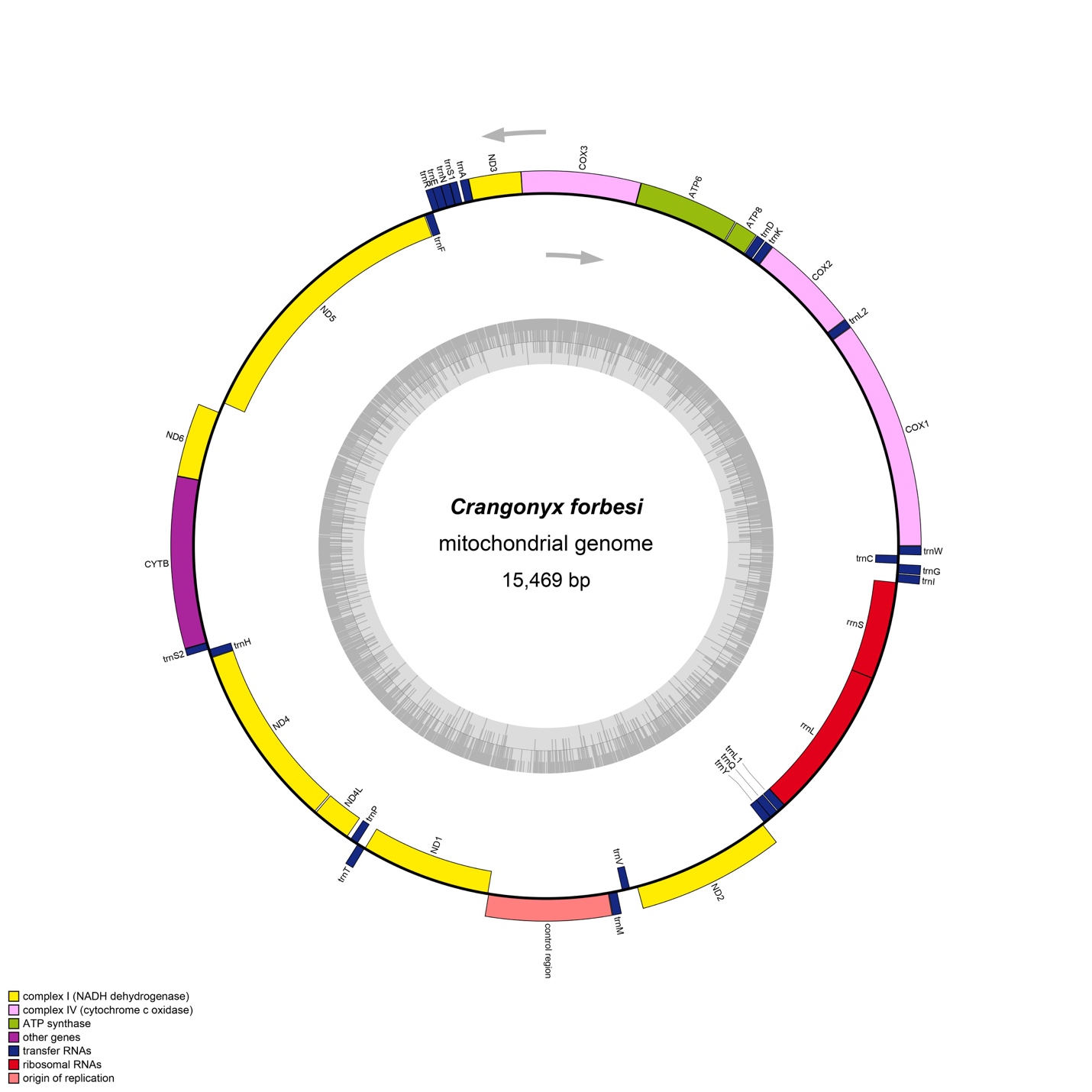
*

**Supplementary Figure S2**. Map of the mitochondrial genome of *S. pizzinii, S. tenuis potomacus, Stygobromus allegheniensis, Bactrurus brachycaudus,* and *Crangonyx forbesi*

**
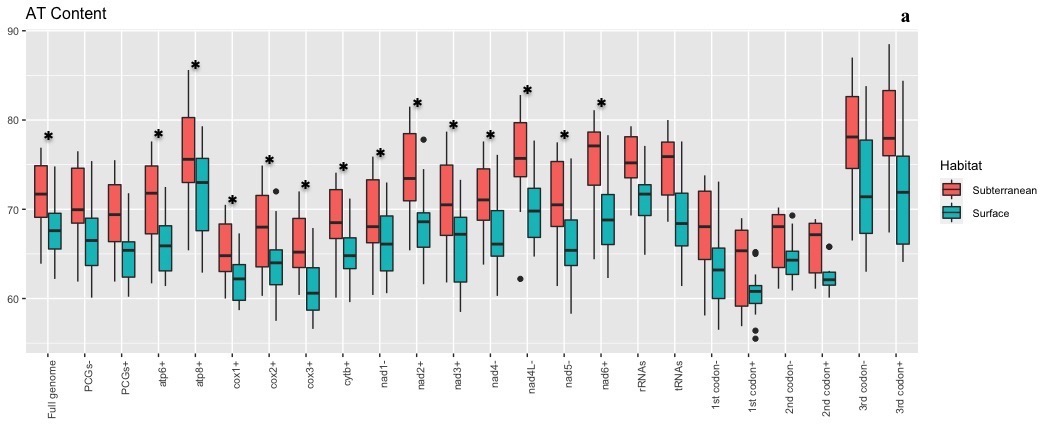
**

**
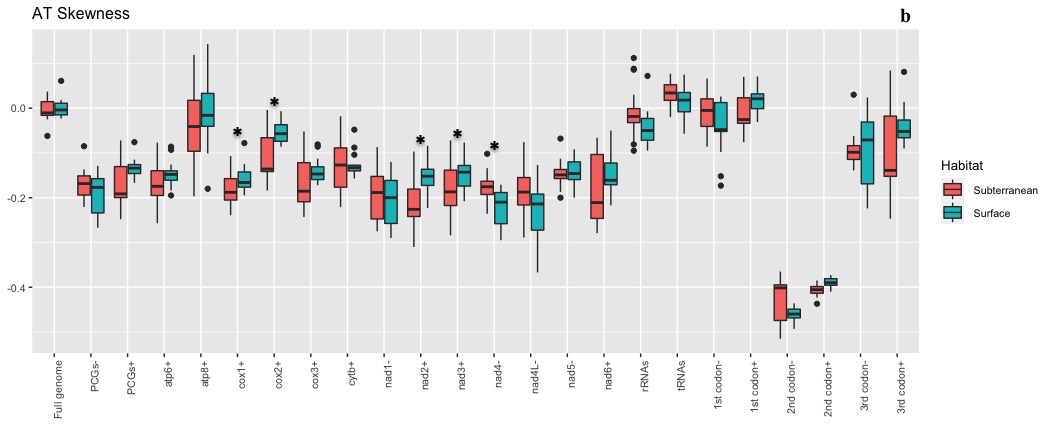
**

**
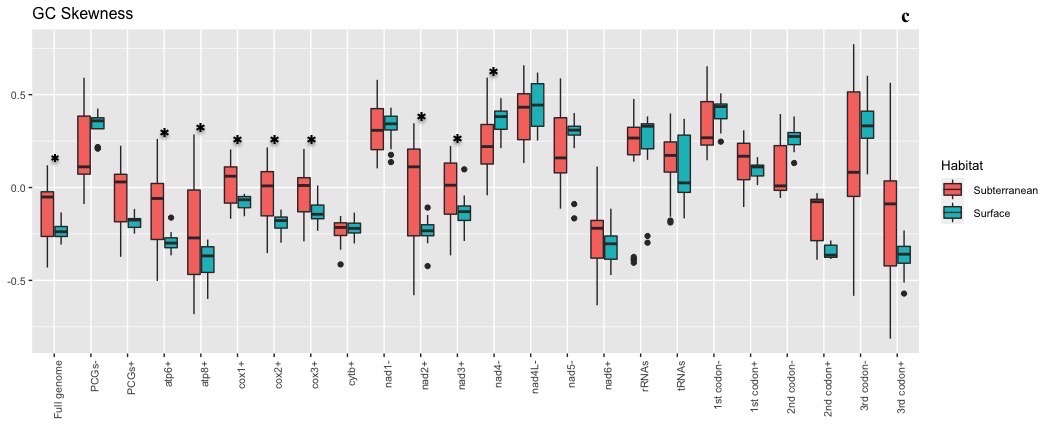
**

**Supplementary Figure S3.** Box plot showing AT percentage **(a)**, AT-skew **(b)**, and GC-skew **(c)** between subterranean and surface amphipods across mitogenomes, protein coding genes (PCG), ribosomal (rRNA) loci, and transfer ribosomal (tRNA) loci. The same features are shown for each protein-coding gene and pooled by codon position and coding strand. Genes coded on the (-) strand are represented by a “-“ sign and genes coded on the (+) strand are represented by “+” sign at the end of the gene label. Mitogenome and PCG with significant P-value < 0.05 are indicated using *.


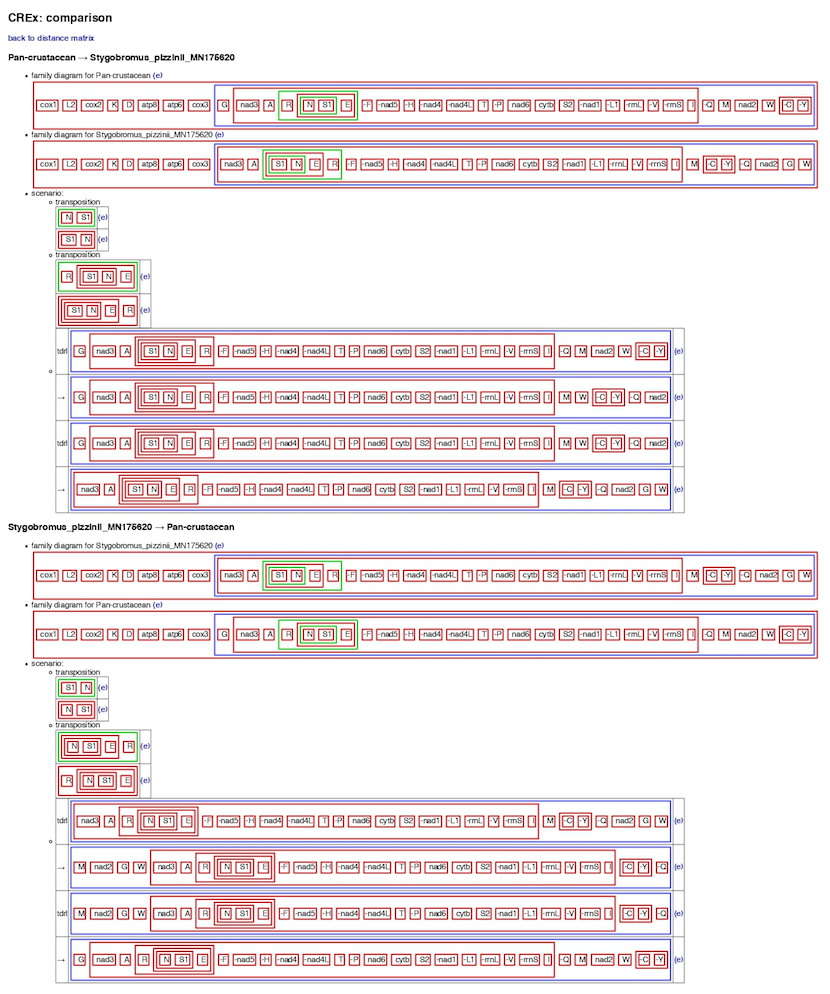


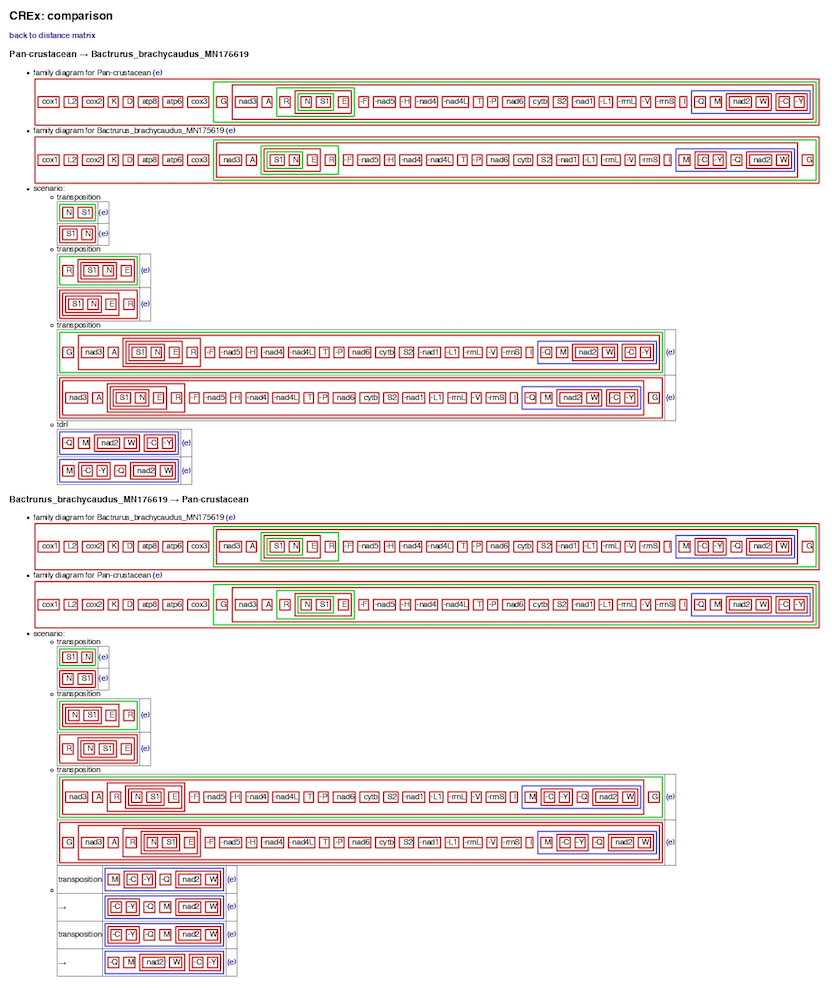


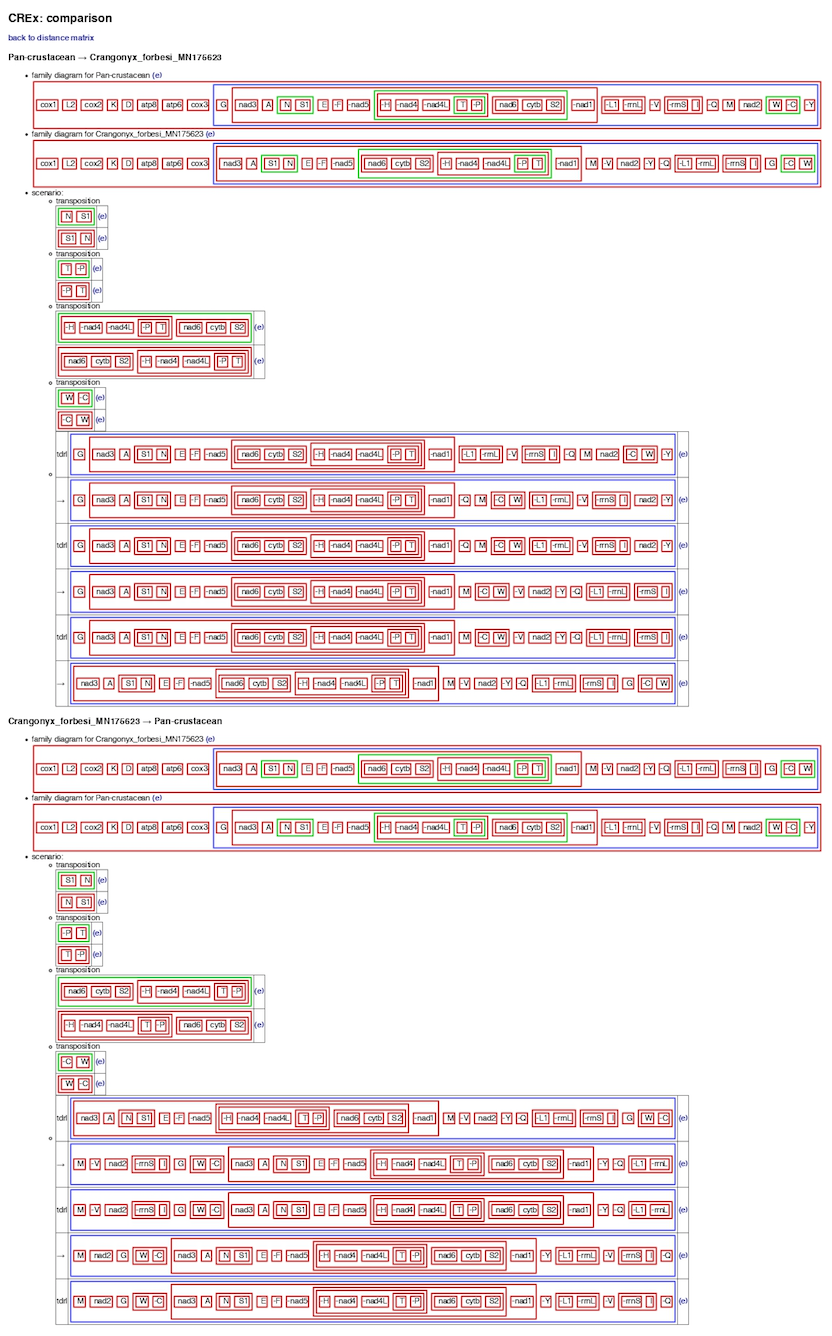


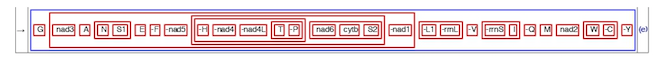


**Supplementary Figure S4**. CREx analysis showing the possible scenarios for the evolution of gene rearrangements in the crangonyctid amphipod genus *Stygobromus*, *Bactrurus*, and *Crangonyx* from the ancestral pan-crustacean pattern


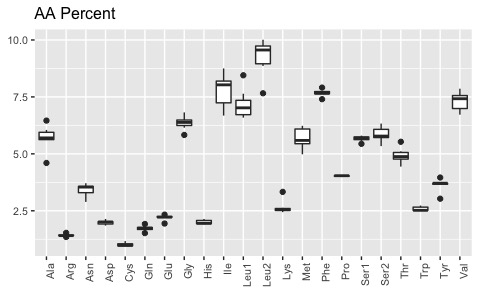


**Supplementary Figure S5.** Box plot showing amino acid composition for mitochondrial protein-coding gene across crangonyctid mitogenomes.


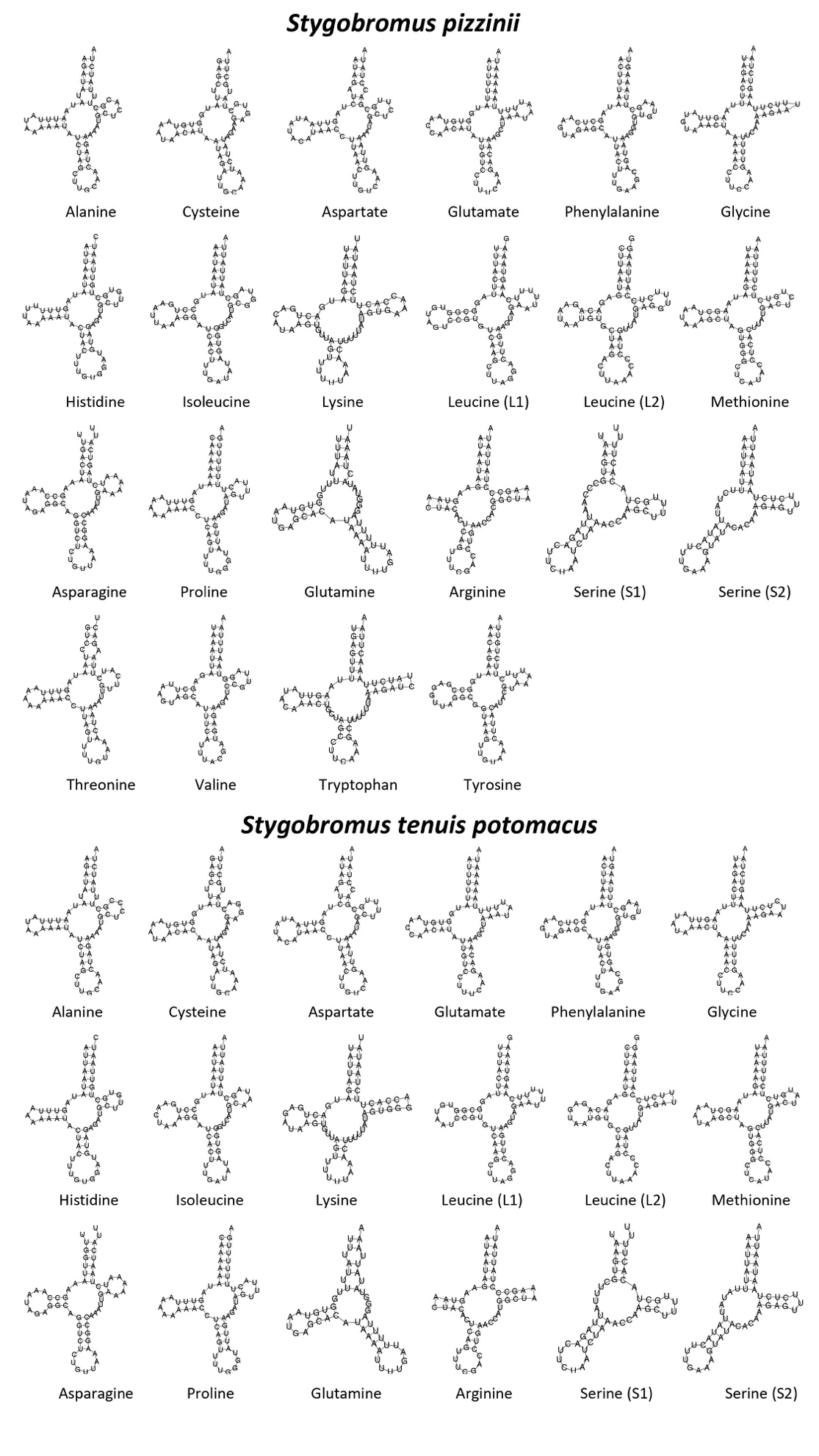


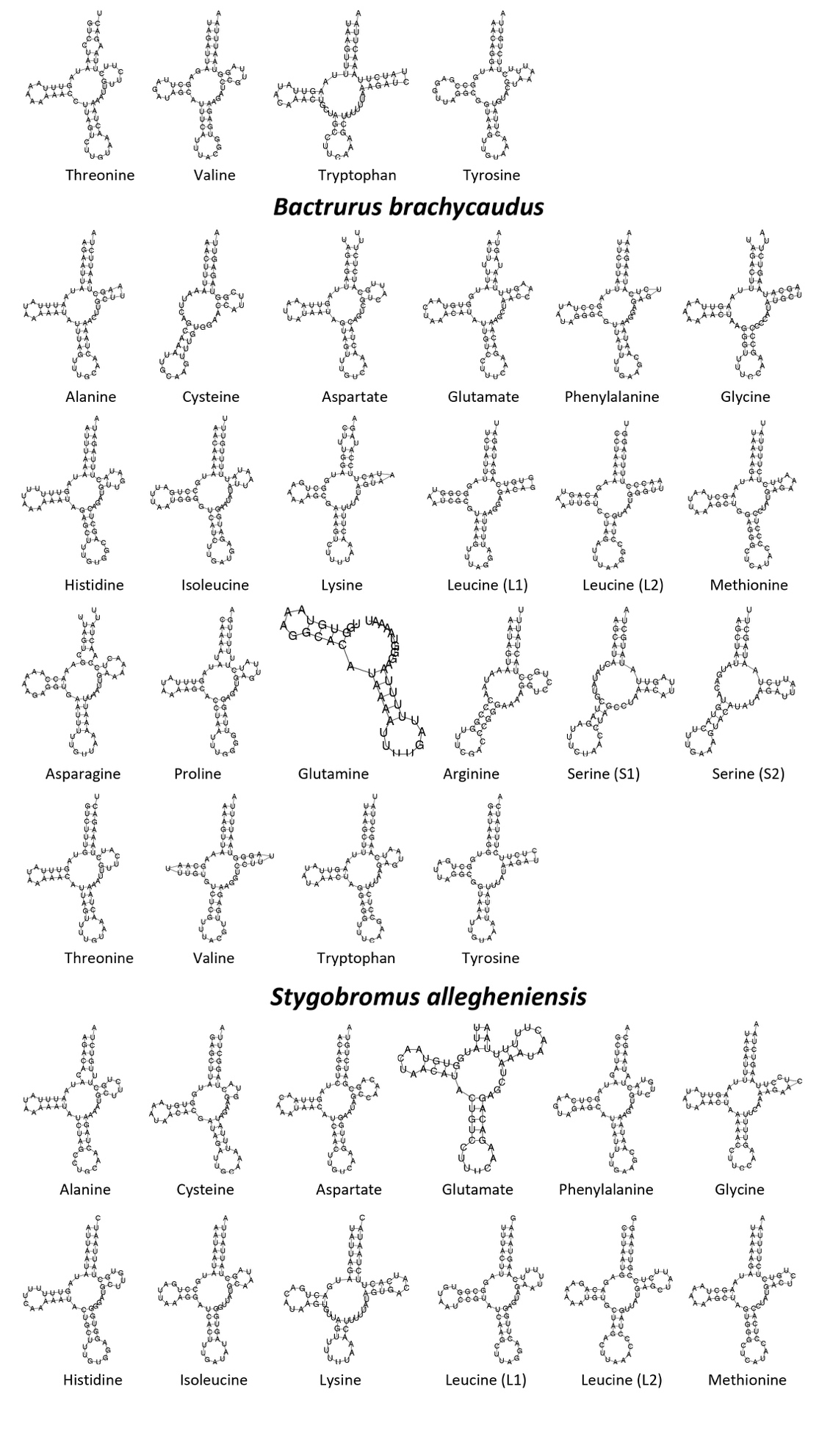


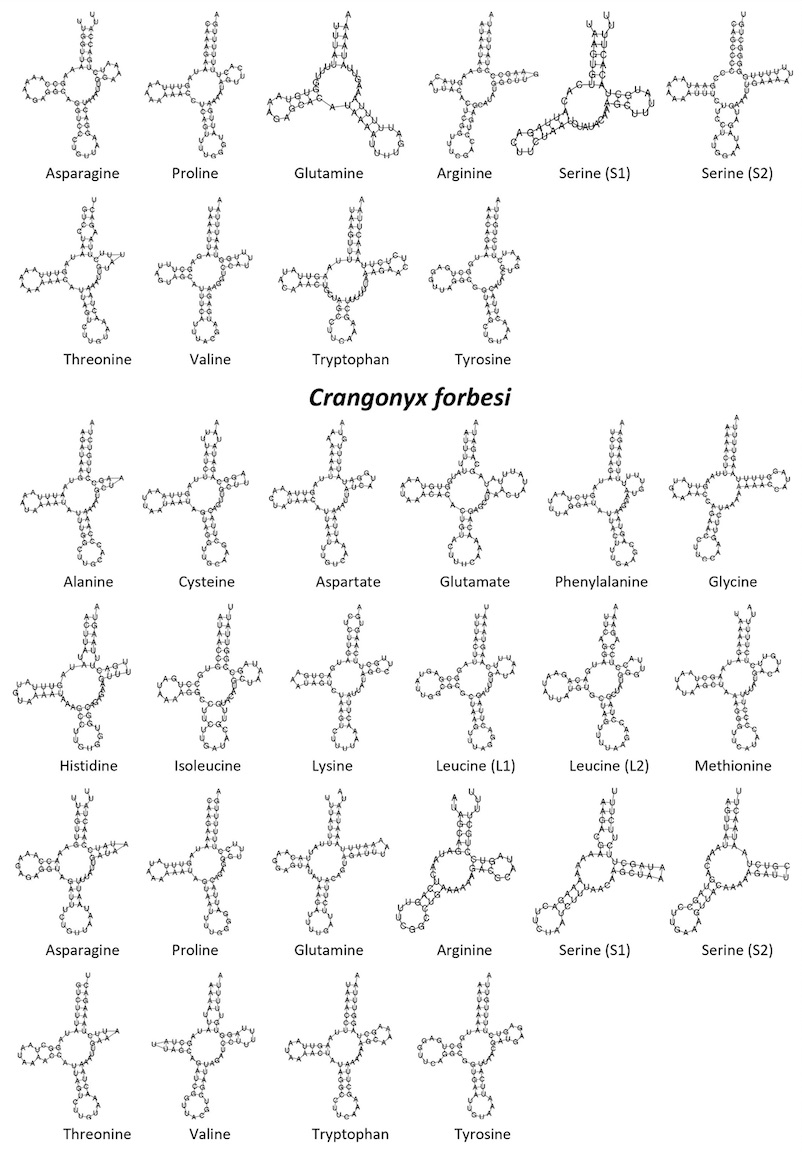


**Supplementary Figure S6.** The predicted mitochondrial tRNAs secondary structures of crangonyctid amphipods under study.

**Supplementary Table S1.** Gblock parameters used for trimming MAFFT alignment of 38 amphipod mitogenomes.

| **Minimum number of sequences for a conserved position** | **Minimum number of sequences for a flank position** | **Minimum number of contiguous nonconserved positions** | **Minimum length of a block** | **Allowed gap positions** | **Use similarity matrices** | **Characters per line in results and parameters file** |
| --- | --- | --- | --- | --- | --- | --- |
| 20 | 20 | 8 | 10 | with half | yes | 60 |

**Supplementary Table S2.** The start and stop position of each gene partition and the best-fit substitution model predicted by PartitionFinder.

| **Partition names** | **Start-stop** | **Best Model** |
| --- | --- | --- |
| atp6 | 1-193 | MTREV+I+G |
| atp8 | 194-205 | MTREV+I+G |
| cox1 | 206-712 | MTREV+I+G |
| cox2 | 713-929 | MTMAM+I+G |
| cox3 | 930-1191 | MTREV+I+G |
| cytb | 1192-1566 | MTREV+I+G |
| nad1 | 1567-1857 | MTREV+I+G |
| nad2 | 1858-2112 | MTMAM+I+G |
| nad3 | 2113-2204 | MTMAM+I+G |
| nad4 | 2205-2577 | MTREV+I+G |
| nad4L | 2578-2673 | MTREV+G |
| nad5 | 2674-3129 | MTREV+I+G |
| nad6 | 3130-3240 | MTMAM+G |

### Supplementary Table S3. Organization of the mitochondrial genomes of crangonyctid amphipods under study.

| ***Bactrurus brachycaudus*** | | |  |  |  |  |  |
| --- | --- | --- | --- | --- | --- | --- | --- |
| **Gene** | **Position** |  | **Size** | **Intergenic nucleotides** | **Codon** |  |  |
|  | **From** | **To** |  |  | **Start** | **Stop** | **Strand** |
| cox1 | 1 | 1534 | 1534 |  | ATC | T | H |
| *trnL2* | 1535 | 1594 | 60 |  |  |  | H |
| *cox2* | 1595 | 2264 | 670 |  | ATG | T | H |
| trnK | 2265 | 2323 | 59 |  |  |  | H |
| *trnD* | 2320 | 2379 | 60 | -4 |  |  | H |
| *atp8* | 2380 | 2544 | 165 |  | ATC | TAA | H |
| *atp6* | 2544 | 3206 | 663 | -1 | ATG | TAA | H |
| *cox3* | 3207 | 4002 | 796 |  | ATG | T | H |
| *nad3* | 4003 | 4353 | 351 |  | ATC | TAG | H |
| *trnA* | 4352 | 4410 | 59 | -2 |  |  | H |
| *trnS1* | 4412 | 4463 | 52 | 1 |  |  | H |
| *trnN* | 4464 | 4525 | 62 |  |  |  | H |
| trnE | 4523 | 4584 | 62 | -3 |  |  | H |
| *trnR* | 4578 | 4627 | 50 | -7 |  |  | H |
| *trnF* | 4625 | 4683 | 59 | -3 |  |  | L |
| *nad5* | 4684 | 6382 | 1699 |  | ATT | T | L |
| trnH | 6383 | 6443 | 61 |  |  |  | L |
| *nad4* | 6444 | 7755 | 1312 |  | ATC | T | L |
| *nad4L* | 7740 | 8030 | 291 | -16 | ATG | TAG | L |
| *trnT* | 8035 | 8092 | 58 | 4 |  |  | H |
| trnP | 8092 | 8150 | 59 | -1 |  |  | L |
| *nad6* | 8153 | 8683 | 531 | 2 | ATG | TAG | H |
| *cytb* | 8697 | 9794 | 1098 | 13 | ATC | TAG | H |
| S_copy2 | 9793 | 9843 | 51 | -2 |  |  | H |
| *nad1* | 9871 | 10794 | 924 | 27 | TTG | TAA | L |
| *L_copy2* | 10795 | 10854 | 60 |  |  |  | L |
| *rrnL* | 10851 | 11884 | 1034 | -4 |  |  | L |
| *trnV* | 11885 | 11941 | 57 |  |  |  | L |
| rrnS | 11939 | 12609 | 671 | -3 |  |  | L |
| *control region* | 12610 | 13140 | 531 |  |  |  | H |
| *trnI* | 13141 | 13202 | 62 |  |  |  | H |
| trnM | 13206 | 13267 | 62 | 3 |  |  | H |
| *trnC* | 13267 | 13316 | 50 | -1 |  |  | L |
| trnY | 13317 | 13376 | 60 |  |  |  | L |
| *trnQ* | 13377 | 13426 | 50 |  |  |  | L |
| *nad2* | 13427 | 14425 | 999 |  | ATT | TAA | H |
| trnW | 14435 | 14494 | 60 | 9 |  |  | H |
| *trnG* | 14497 | 14558 | 62 | 2 |  |  | H |
| *Overlap:* | 12 | gap: | 8 |  |  |  |  |
|  |  |  |  |  |  |  |  |
| ***Stygobromus pizzinii*** | | |  |  |  |  |  |
| **Gene** | **Position** |  | **Size** | **Intergenic nucleotides** | **Codon** |  |  |
|  | **From** | **To** |  |  | **Start** | **Stop** | **Strand** |
| *cox1* | 1 | 1534 | 1534 |  | ATA | T | H |
| *trnL2* | 1535 | 1596 | 62 |  |  |  | H |
| *cox2* | 1596 | 2270 | 675 | -1 | GTG | TAA | H |
| *trnK* | 2271 | 2333 | 63 |  |  |  | H |
| *trnD* | 2332 | 2393 | 62 | -2 |  |  | H |
| *atp8* | 2394 | 2582 | 189 |  | ATC | TAG | H |
| *atp6* | 2542 | 3210 | 669 | -41 | ATG | TAA | H |
| cox3 | 3210 | 4004 | 795 | -1 | ATG | TAA | H |
| *nad3* | 4036 | 4386 | 351 | 31 | ATT | TAG | H |
| *trnA* | 4385 | 4443 | 59 | -2 |  |  | H |
| *trnS1* | 4444 | 4493 | 50 |  |  |  | H |
| trnN | 4492 | 4553 | 62 | -2 |  |  | H |
| *trnE* | 4551 | 4612 | 62 | -3 |  |  | H |
| *trnR* | 4610 | 4666 | 57 | -3 |  |  | H |
| *trnF* | 4665 | 4724 | 60 | -2 |  |  | L |
| *nad5* | 4725 | 6432 | 1708 |  | ATT | T | L |
| *trnH* | 6433 | 6492 | 60 |  |  |  | L |
| *nad4* | 6493 | 7813 | 1321 |  | ATG | T | L |
| *nad4L* | 7807 | 8100 | 294 | -7 | ATG | TAA | L |
| *trnT* | 8104 | 8162 | 59 | 3 |  |  | H |
| trnP | 8162 | 8221 | 60 | -1 |  |  | L |
| *nad6* | 8226 | 8726 | 501 | 4 | ATG | TAA | H |
| *cytb* | 8726 | 9865 | 1140 | -1 | ATG | TAA | H |
| S_copy2 | 9864 | 9916 | 53 | -2 |  |  | H |
| *nad1* | 9937 | 10857 | 921 | 20 | TTG | TAA | L |
| *L_copy2* | 10895 | 10956 | 62 | 37 |  |  | L |
| *rrnL* | 10957 | 11993 | 1037 |  |  |  | L |
| trnV | 11994 | 12051 | 58 |  |  |  | L |
| *rrnS* | 12052 | 12729 | 678 |  |  |  | L |
| *control region* | 12730 | 13750 | 1021 |  |  |  | H |
| *trnI* | 13751 | 13811 | 61 |  |  |  | H |
| *trnM* | 13815 | 13875 | 61 | 3 |  |  | H |
| *trnC* | 13873 | 13930 | 58 | -3 |  |  | L |
| *trnY* | 13931 | 13991 | 61 |  |  |  | L |
| *trnQ* | 13993 | 14044 | 52 | 1 |  |  | L |
| *nad2* | 14053 | 15046 | 994 | 8 | ATA | T | H |
| trnG | 15047 | 15109 | 63 |  |  |  | H |
| *trnW* | 15110 | 15173 | 64 |  |  |  | H |
| Overlap: | 14 | gap: | 8 |  |  |  |  |
|  |  |  |  |  |  |  |  |
| ***Stygobromus tenuis potomacus*** | | |  |  |  |  |  |
| **Gene** | **Position** |  | **Size** | **Intergenic nucleotides** | **Codon** |  |  |
|  | **From** | **To** |  |  | **Start** | **Stop** | **Strand** |
| *cox1* | 1 | 1534 | 1534 |  | ATG | T | H |
| *trnL2* | 1535 | 1595 | 61 |  |  |  | H |
| *cox2* | 1596 | 2270 | 675 |  | GTG | TAA | H |
| *trnK* | 2271 | 2333 | 63 |  |  |  | H |
| *trnD* | 2332 | 2393 | 62 | -2 |  |  | H |
| *atp8* | 2394 | 2582 | 189 |  | ATT | TAG | H |
| *atp6* | 2542 | 3210 | 669 | -41 | ATG | TAA | H |
| cox3 | **3210** | 4004 | 795 | -1 | ATG | TAA | H |
| *nad3* | 4034 | 4387 | 354 | 29 | ATT | TAG | H |
| *trnA* | 4386 | 4444 | 59 | -2 |  |  | H |
| *trnS1* | 4445 | 4494 | 50 |  |  |  | H |
| trnN | **4493** | 4554 | 62 | -2 |  |  | H |
| *trnE* | 4552 | 4612 | 61 | -3 |  |  | H |
| trnR | **4610** | 4666 | 57 | -3 |  |  | H |
| *trnF* | 4665 | 4724 | 60 | -2 |  |  | L |
| *nad5* | 4725 | 6432 | 1708 |  | ATG | T | L |
| *trnH* | 6433 | 6490 | 58 |  |  |  | L |
| *nad4* | 6491 | 7811 | 1321 |  | ATG | T | L |
| *nad4L* | 7805 | 8098 | 294 | -7 | ATG | TAA | L |
| *trnT* | 8102 | 8160 | 59 | 3 |  |  | H |
| *trnP* | 8160 | 8219 | 60 | -1 |  |  | L |
| *nad6* | 8223 | 8723 | 501 | 3 | ATG | TAA | H |
| *cytb* | 8723 | 9862 | 1140 | -1 | ATG | TAA | H |
| *S_copy2* | 9861 | 9913 | 53 | -2 |  |  | H |
| *nad1* | 9935 | 10855 | 921 | 21 | TTG | TAG | L |
| *L_copy2* | 10891 | 10953 | 63 | 35 |  |  | L |
| rrnL | **10954** | 11989 | 1036 |  |  |  | L |
| *trnV* | 11990 | 12047 | 58 |  |  |  | L |
| *rrnS* | 12048 | 12726 | 679 |  |  |  | L |
| *control region* | 12727 | 13282 | 556 |  |  |  | H |
| trnI | **13283** | 13343 | 61 |  |  |  | H |
| *trnM* | 13347 | 13407 | 61 | 3 |  |  | H |
| *trnC* | 13405 | 13462 | 58 | -3 |  |  | L |
| *trnY* | 13463 | 13523 | 61 |  |  |  | L |
| *trnQ* | 13525 | 13576 | 52 | 1 |  |  | L |
| *nad2* | 13589 | 14582 | 994 | 12 | ATA | T | H |
| *trnG* | 14583 | 14645 | 63 |  |  |  | H |
| trnW | 14646 | 14709 | 64 |  |  |  | H |
| *Overlap:* | 13 | gap: | 8 |  |  |  |  |
|  |  |  |  |  |  |  |  |
| ***Stygobromus allegheniensis*** | | |  |  |  |  |  |
| **Gene** | **Position** |  | **Size** | **Intergenic nucleotides** | **Codon** |  |  |
|  | **From** | **To** |  |  | **Start** | **Stop** | **Strand** |
| cox1 | 1 | 1534 | 1534 |  | ATC | T | H |
| *trnL2* | 1535 | 1596 | 62 |  |  |  | H |
| *cox2* | 1597 | 2271 | 675 |  | ATG | TAG | H |
| *trnK* | 2272 | 2334 | 63 |  |  |  | H |
| *trnD* | 2333 | 2393 | 61 | -2 |  |  | H |
| *atp8* | 2403 | 2582 | 180 | 9 | ATA | TAA | H |
| *atp6* | 2542 | 3210 | 669 | -41 | ATG | TAA | H |
| *cox3* | 3210 | 4002 | 793 | -1 | ATG | T | H |
| nad3 | 4031 | 4384 | 354 | 28 | ATT | TAA | H |
| *trnA* | 4386 | 4444 | 59 | 1 |  |  | H |
| trnS1 | 4445 | 4495 | 51 |  |  |  | H |
| *trnN* | 4494 | 4554 | 61 | -2 |  |  | H |
| trnE | 4556 | 4610 | 55 | 1 |  |  | H |
| *trnR* | 4611 | 4670 | 60 |  |  |  | H |
| trnF | 4673 | 4732 | 60 | 2 |  |  | L |
| *nad5* | 4733 | 6440 | 1708 |  | GTG | T | L |
| *trnH* | 6441 | 6501 | 61 |  |  |  | L |
| nad4 | 6502 | 7822 | 1321 |  | ATG | T | L |
| *nad4L* | 7816 | 8109 | 294 | -7 | ATG | TAG | L |
| *trnT* | 8113 | 8172 | 60 | 3 |  |  | H |
| *trnP* | 8172 | 8231 | 60 | -1 |  |  | L |
| nad6 | 8235 | 8735 | 501 | 3 | ATG | TAA | H |
| *cytb* | 8735 | 9874 | 1140 | -1 | ATG | TAA | H |
| S_copy2 | 9873 | 9926 | 54 | -2 |  |  | H |
| *nad1* | 9949 | 10869 | 921 | 22 | TTG | TAG | L |
| L_copy2 | 10904 | 10965 | 62 | 34 |  |  | L |
| *rrnL* | 10967 | 12000 | 1034 | 1 |  |  | L |
| trnV | 12002 | 12060 | 59 | 1 |  |  | L |
| *rrnS* | 12061 | 12748 | 688 |  |  |  | L |
| *control region* | 12749 | 13739 | 991 |  |  |  | H |
| trnI | 13740 | 13799 | 60 |  |  |  | H |
| *trnM* | 13802 | 13862 | 61 | 2 |  |  | H |
| trnC | 13860 | 13916 | 57 | -3 |  |  | L |
| *trnY* | 13918 | 13976 | 59 | 1 |  |  | L |
| trnQ | 13979 | 14029 | 51 | 2 |  |  | L |
| *nad2* | 14041 | 14935 | 895 | 11 | ATA | T | H |
| *trnG* | 15035 | 15097 | 63 | 99 |  |  | H |
| trnW | 15098 | 15161 | 64 |  |  |  | H |
| *Overlap:* | 9 | gap: | 16 |  |  |  |  |
|  |  |  |  |  |  |  |  |
| ***Crangonyx forbesi*** | |  |  |  |  |  |  |
| **Gene** | **Position** |  | **Size** | **Intergenic nucleotides** | **Codon** |  |  |
|  | **From** | **To** |  |  | **Start** | **Stop** | **Strand** |
| *cox1* | 1 | 1534 | 1534 |  | ATT | T | H |
| trnL2 | 1535 | 1595 | 61 |  |  |  | H |
| *cox2* | 1596 | 2271 | 676 |  | ATT | T | H |
| trnK | 2272 | 2330 | 59 |  |  |  | H |
| *trnD* | 2337 | 2397 | 61 | 6 |  |  | H |
| *atp8* | 2401 | 2559 | 159 | 3 | ATG | TAA | H |
| *atp6* | 2564 | 3235 | 672 | 4 | ATG | TAA | H |
| *cox3* | 3235 | 4033 | 799 | -1 | ATG | T | H |
| *nad3* | 4031 | 4381 | 351 | -3 | ATT | TAG | H |
| *trnA* | 4380 | 4438 | 59 | -2 |  |  | H |
| *trnS1* | 4456 | 4507 | 52 | 17 |  |  | H |
| *trnN* | 4507 | 4568 | 62 | -1 |  |  | H |
| trnE | 4566 | 4631 | 66 | -3 |  |  | H |
| *trnR* | 4622 | 4672 | 51 | -10 |  |  | H |
| *trnF* | 4671 | 4730 | 60 | -2 |  |  | L |
| nad5 | 4734 | 6707 | 1974 | 3 | ATT | TAA | L |
| *nad6* | 6779 | 7273 | 495 | 71 | ATG | TAA | H |
| *cytb* | 7273 | 8418 | 1146 | -1 | ATG | TAA | H |
| S_copy2 | 8418 | 8467 | 50 | -1 |  |  | H |
| *trnH* | 8467 | 8526 | 60 | -1 |  |  | L |
| *nad4* | 8527 | 9838 | 1312 |  | ATG | T | L |
| nad4L | 9847 | 10125 | 279 | 8 | ATA | TAA | L |
| *trnP* | 10153 | 10211 | 59 | 27 |  |  | L |
| *trnT* | 10214 | 10272 | 59 | 2 |  |  | H |
| nad1 | 10272 | 11195 | 924 | -1 | GTG | TAA | L |
| *control region* | 11198 | 12040 | 843 | 2 |  |  | H |
| *trnM* | 12041 | 12102 | 62 |  |  |  | H |
| trnV | 12137 | 12194 | 58 | 34 |  |  | L |
| *nad2* | 12245 | 13231 | 987 | 50 | ATA | TAA | H |
| *trnY* | 13245 | 13307 | 63 | 13 |  |  | L |
| trnQ | 13304 | 13368 | 65 | -4 |  |  | L |
| *L_copy2* | 13372 | 13433 | 62 | 3 |  |  | L |
| rrnL | 13433 | 14522 | 1090 | -1 |  |  | L |
| *rrnS* | 14521 | 15215 | 695 | -2 |  |  | L |
| trnI | 15216 | 15274 | 59 |  |  |  | H |
| *trnG* | 15278 | 15343 | 66 | 3 |  |  | H |
| trnC | 15345 | 15405 | 61 | 1 |  |  | L |
| *trnW* | 15408 | 15469 | 62 | 2 |  |  | H |
| Overlap: | 14 | gap: | 17 |  |  |  |  |

**Supplementary Table S4.** Summary of putative start codons in PCG of mitochondrial genomes of all amphipods

|  |  | **Putative start codon** | | | | | | | | | | | | |
| --- | --- | --- | --- | --- | --- | --- | --- | --- | --- | --- | --- | --- | --- | --- |
| **Species** | **Accession** | ***atp6*** | ***atp8*** | ***cox1*** | ***cox2*** | ***cox3*** | ***cytb*** | ***nad1*** | ***nad2*** | ***nad3*** | ***nad4*** | ***nad4L*** | ***nad5*** | ***nad6*** |
| *Stygobromus pizzinii* | MN175620 | ATG | ATC | ATA | GTG | ATG | ATG | TTG | ATA | ATT | ATG | ATG | ATT | ATG |
| *Stygobromus tenuis potomacus* | MN175621 | ATG | ATT | ATG | GTG | ATG | ATG | TTG | ATA | ATT | ATG | ATG | ATG | ATG |
| *Bactrurus brachycaudus* | MN175619 | ATG | ATC | ATC | ATG | ATG | ATC | TTG | ATT | ATC | ATC | ATG | ATT | ATG |
| *Stygobromus allegheniensis* | MN175622 | ATG | GTA | ATC | ATG | ATG | ATG | TTG | ATA | ATT | ATG | ATG | GTG | ATG |
| *Crangonyx forbesi* | MN175623 | ATA | ATG | ATT | ATT | ATG | ATG | GTG | ATT | ATT | ATG | ATA | ATT | ATG |
| *Gondogeneia antarctica* | JN827386.1 | ATG | ATT | ATG | ATA | ATG | ATG | ATT | ATT | ATA | ATG | ATG | ATT | ATG |
| *Gmelinoides fasciatus* | NC_033361.1 | ATG | ATC | ATA | TTG | ATG | ATG | ATA | TTG | ATG | GTG | ATG | ATG | ATG |
| *Brachyuropus grewingkii* | NC_026309.1 | ATG | GTG | ATT | TTG | ATG | ATG | TTG | TTG | ATG | ATG | ATG | ATT | GTG |
| *Gammarus fossarum* | NC_034937.1 | ATA | ATC | ATA | TTG | ATG | ATA | TTG | TTG | ATG | ATA | ATG | TTG | ATG |
| *Pallaseopsis kessleri* | NC_033362.1 | ATG | ATT | ATT | GTG | ATG | ATG | TTG | TTG | ATG | TTG | ATG | TTG | ATT |
| *Gammarus duebeni* | NC_017760.1 | ATG | ATA | ATA | TTG | ATG | ATG | ATA | TTG | ATG | ATA | ATG | GTG | ATG |
| *Eulimnogammarus vittatus* | NC_025564.1 | ATG | GTG | ATT | TTG | ATG | ATG | TTG | TTG | ATG | ATG | ATG | TTG | ATG |
| *Caprella mutica* | NC_014492.1 | ATG | ATA | ATT | ATA | ATA | ATG | ATA | ATT | ATG | ATG | TTG | ATA | ATT |
| *Eulimnogammarus verrucosus* | NC_023104.1 | ATG | GTG | ATT | TTG | ATG | ATG | ATT | TTG | ATG | ATA | ATA | ATA | ATG |
| *Pseudoniphargus daviui* | NC_019662.2 | ATG | ATA | ATT | TTG | ATG | ATG | ATA | ATC | TTG | ATA | ATG | TTG | AAT |
| *Caprella scaura* | NC_014687.1 | ATG | ATA | ATT | ATA | ATG | ATG | CTG | ATG | ATG | ATG | CTG | ATA | ATA |
| *Metacrangonyx spinicaudatus* | NC_019657.1 | ATG | ATA | ATT | ATC | ATG | ATG | ATA | ATT | ATT | ATG | ATG | TTG | ATT |
| *Stygobromus tenuis potomacus* | KU869712.1 | ATG | ATT | ATT | ATC | ATG | ATG | TTG | ATA | ATA | ATG | ATG | ATA | ATG |
| *Metacrangonyx remyi* | NC_019660.1 | ATG | GTG | ATC | ATT | ATG | ATG | ATA | ATA | ATT | ATG | ATG | CTG | ATT |
| *Platorchestia parapacifica* | MG010371.1 | ATA | ATG | ATT | ATT | ATG | ATA | ATT | ATC | ATG | ATG | ATT | ATA | ATC |
| *Platorchestia japonica* | MG010370.1 | ATA | ATG | ATT | ATA | ATG | ATA | ATT | ATA | ATG | ATG | ATA | ATA | ATT |
| *Metacrangonyx ilvanus* | NC_019656.1 | ATG | ATC | ATT | ATT | ATG | ATG | ATA | ATT | ATT | ATG | ATG | TTG | ATT |
| *Onisimus nanseni* | NC_013819.1 | ATG | ATT | ATG | TTG | ATG | ATG | ATA | ATG | ATG | TTG | ATG | GTG | ATT |
| *Metacrangonyx longicaudus* | NC_019658.1 | ATG | ATT | ATT | ATC | ATG | ATG | ATA | ATT | ATT | ATG | ATG | TTG | ATT |
| *Bahadzia jaraguensis* | FR872382.1 | ATG | ATC | ATT | ATT | ATG | ATG | ATG | ATT | ATT | ATG | ATG | ATA | ATT |
| *Stygobromus indentatus* | NC_030261.1 | ATA | ATT | ATT | ATA | ATG | ATG | GTG | ATT | ATA | ATT | ATG | GTG | ATG |
| *Metacrangonyx dominicanus* | NC_019654.1 | ATG | ATC | ATT | ATT | ATG | ATG | ATA | ATT | ATC | ATG | ATG | TTG | ATT |
| *Metacrangonyx goulmimensis* | NC_019655.1 | ATG | ATC | ATT | ATC | ATG | ATG | ATA | ATT | ATC | ATG | ATG | TTG | ATT |
| *Metacrangonyx panousei* | NC_019659.1 | ATG | ATC | ATT | ATT | ATG | ATG | ATA | ATT | ATT | ATG | ATG | TTG | ATT |
| *Eulimnogammarus cyaneus* | NC_033360.1 | ATG | GTG | ATC | TTG | ATG | ATG | TTG | TTG | ATG | ATG | ATG | GTG | ATG |
| *Metacrangonyx repens* | NC_019653.1 | ATG | GTG | ATT | ATT | ATG | ATG | ATA | ATT | ATT | ATG | ATG | TTG | ATT |
| *Metacrangonyx longipes* | NC_013032.1 | ATG | ATC | ATT | ATT | ATG | ATG | ATA | ATT | ATT | ATA | ATA | ATT | ATT |
| *Metacrangonyx sp. 3 ssp. 1 MDMBR-2012* | HE860504.1 | ATG | ATC | ATT | ATT | ATG | ATG | ATA | ATT | ATT | ATG | ATG | TTG | ATT |
| *Metacrangonyx sp. 4 MDMBR-2012* | HE860498.1 | ATG | ATC | ATC | ATC | ATG | ATG | ATA | ATA | ATC | GTG | ATG | TTG | ATT |
| *Metacrangonyx sp. 1 MDMBR-2012* | HE860513.1 | ATG | ATC | ATT | ATC | ATG | ATG | ATA | TTT | ATT | ATG | ATG | TTG | ATT |

**Supplementary Table S5.** Summary of codon usage in PCG of mitochondrial genomes of all crangonyctid amphipods. Sign “#” indicates total number of certain codon in protein-coding sequences of every species; “%” indicates percent of certain codon in total coding sequence in every species; RSCU indicates the calculated RSCU value of certain codon in total coding sequence in every species

| **AA** | **Codon** | **Stygobromus indentatus** | | | **Crangonyx forbesi** | | | **Stygobromus allegheniensis** | | | **Stygobromus tenuis potomacus** | | | **Stygobromus pizzinii** | | | **Bactrurus brachycaudus** | | | **Stygobromus tenuis potomacus** | | |
| --- | --- | --- | --- | --- | --- | --- | --- | --- | --- | --- | --- | --- | --- | --- | --- | --- | --- | --- | --- | --- | --- | --- |
|  |  | **RSCU** | **#** | **%** | **RSCU** | **#** | **%** | **RSCU** | **#** | **%** | **RSCU** | **#** | **%** | **RSCU** | **#** | **%** | **RSCU** | **#** | **%** | **RSCU** | **#** | **%** |
| Phe | UUU | 1.74 | 238 | 6.45 | 1.76 | 255 | 6.78 | 1.64 | 237 | 6.49 | 1.70 | 240 | 6.51 | 1.69 | 241 | 6.54 | 1.55 | 218 | 5.94 | 1.72 | 243 | 6.57 |
|  | UUC | 0.26 | 35 | 0.95 | 0.24 | 35 | 0.93 | 0.36 | 52 | 1.42 | 0.30 | 42 | 1.14 | 0.31 | 45 | 1.22 | 0.45 | 63 | 1.72 | 0.28 | 39 | 1.05 |
| Leu2 | UUA | 2.89 | 286 | 7.75 | 2.58 | 267 | 7.10 | 2.56 | 259 | 7.09 | 2.95 | 298 | 8.08 | 2.96 | 297 | 8.06 | 2.10 | 207 | 5.64 | 3.04 | 314 | 8.49 |
|  | UUG | 0.49 | 48 | 1.30 | 0.64 | 66 | 1.76 | 0.89 | 90 | 2.46 | 0.64 | 65 | 1.76 | 0.58 | 58 | 1.57 | 0.75 | 74 | 2.02 | 0.54 | 56 | 1.51 |
| Leu1 | CUU | 1.00 | 99 | 2.68 | 1.05 | 109 | 2.90 | 0.97 | 98 | 2.68 | 1.02 | 103 | 2.79 | 1.03 | 103 | 2.79 | 1.18 | 116 | 3.16 | 0.99 | 102 | 2.76 |
|  | CUC | 0.33 | 33 | 0.89 | 0.32 | 33 | 0.88 | 0.27 | 27 | 0.74 | 0.26 | 26 | 0.70 | 0.24 | 24 | 0.65 | 0.46 | 45 | 1.23 | 0.27 | 28 | 0.76 |
|  | CUA | 1.10 | 109 | 2.95 | 1.07 | 111 | 2.95 | 0.99 | 100 | 2.74 | 0.88 | 89 | 2.41 | 0.97 | 97 | 2.63 | 1.12 | 110 | 3.00 | 0.91 | 94 | 2.54 |
|  | CUG | 0.18 | 18 | 0.49 | 0.33 | 34 | 0.90 | 0.33 | 33 | 0.90 | 0.25 | 25 | 0.68 | 0.23 | 23 | 0.62 | 0.40 | 39 | 1.06 | 0.24 | 25 | 0.68 |
| Ile | AUU | 1.57 | 253 | 6.85 | 1.47 | 185 | 4.92 | 1.45 | 188 | 5.15 | 1.62 | 240 | 6.51 | 1.61 | 245 | 6.64 | 1.48 | 201 | 5.48 | 1.53 | 230 | 6.22 |
|  | AUC | 0.43 | 70 | 1.90 | 0.53 | 66 | 1.76 | 0.55 | 71 | 1.94 | 0.38 | 56 | 1.52 | 0.39 | 59 | 1.60 | 0.52 | 70 | 1.91 | 0.47 | 71 | 1.92 |
| Met | AUA | 1.71 | 197 | 5.34 | 1.61 | 183 | 4.87 | 1.66 | 151 | 4.13 | 1.64 | 169 | 4.58 | 1.66 | 169 | 4.58 | 1.37 | 153 | 4.17 | 1.70 | 168 | 4.54 |
|  | AUG | 0.29 | 33 | 0.89 | 0.39 | 45 | 1.20 | 0.34 | 31 | 0.85 | 0.36 | 37 | 1.00 | 0.34 | 35 | 0.95 | 0.63 | 71 | 1.94 | 0.30 | 30 | 0.81 |
| Val | GUU | 1.71 | 106 | 2.87 | 1.61 | 112 | 2.98 | 1.55 | 111 | 3.04 | 1.72 | 118 | 3.20 | 1.57 | 101 | 2.74 | 1.36 | 96 | 2.62 | 1.60 | 103 | 2.79 |
|  | GUC | 0.32 | 20 | 0.54 | 0.46 | 32 | 0.85 | 0.50 | 36 | 0.99 | 0.42 | 29 | 0.79 | 0.50 | 32 | 0.87 | 0.54 | 38 | 1.04 | 0.34 | 22 | 0.60 |
|  | GUA | 1.24 | 77 | 2.09 | 1.38 | 96 | 2.55 | 1.17 | 84 | 2.30 | 1.39 | 95 | 2.58 | 1.40 | 90 | 2.44 | 1.52 | 107 | 2.92 | 1.61 | 104 | 2.81 |
|  | GUG | 0.73 | 45 | 1.22 | 0.56 | 39 | 1.04 | 0.78 | 56 | 1.53 | 0.47 | 32 | 0.87 | 0.54 | 35 | 0.95 | 0.58 | 41 | 1.12 | 0.45 | 29 | 0.78 |
| Ser2 | UCU | 2.01 | 108 | 2.93 | 1.93 | 110 | 2.93 | 1.81 | 92 | 2.52 | 1.80 | 94 | 2.55 | 1.89 | 100 | 2.71 | 1.74 | 95 | 2.59 | 1.95 | 100 | 2.70 |
|  | UCC | 0.56 | 30 | 0.81 | 0.47 | 27 | 0.72 | 0.47 | 24 | 0.66 | 0.63 | 33 | 0.89 | 0.43 | 23 | 0.62 | 0.75 | 41 | 1.12 | 0.25 | 13 | 0.35 |
|  | UCA | 1.34 | 72 | 1.95 | 1.39 | 79 | 2.10 | 1.38 | 70 | 1.92 | 1.45 | 76 | 2.06 | 1.61 | 85 | 2.31 | 1.37 | 75 | 2.04 | 1.74 | 89 | 2.41 |
|  | UCG | 0.17 | 9 | 0.24 | 0.39 | 22 | 0.59 | 0.18 | 9 | 0.25 | 0.19 | 10 | 0.27 | 0.09 | 5 | 0.14 | 0.31 | 17 | 0.46 | 0.14 | 7 | 0.19 |
| Pro | CCU | 1.88 | 69 | 1.87 | 1.87 | 71 | 1.89 | 1.95 | 71 | 1.94 | 1.66 | 62 | 1.68 | 1.72 | 64 | 1.74 | 1.76 | 65 | 1.77 | 1.79 | 67 | 1.81 |
|  | CCC | 0.54 | 20 | 0.54 | 0.84 | 32 | 0.85 | 0.44 | 16 | 0.44 | 0.27 | 10 | 0.27 | 0.56 | 21 | 0.57 | 0.65 | 24 | 0.65 | 0.61 | 23 | 0.62 |
|  | CCA | 1.31 | 48 | 1.30 | 0.84 | 32 | 0.85 | 1.37 | 50 | 1.37 | 1.80 | 67 | 1.82 | 1.40 | 52 | 1.41 | 1.11 | 41 | 1.12 | 1.44 | 54 | 1.46 |
|  | CCG | 0.27 | 10 | 0.27 | 0.45 | 17 | 0.45 | 0.25 | 9 | 0.25 | 0.27 | 10 | 0.27 | 0.32 | 12 | 0.33 | 0.49 | 18 | 0.49 | 0.16 | 6 | 0.16 |
| Thr | ACU | 1.59 | 70 | 1.90 | 1.42 | 74 | 1.97 | 1.81 | 83 | 2.27 | 1.70 | 75 | 2.03 | 1.76 | 79 | 2.14 | 1.37 | 56 | 1.53 | 1.67 | 79 | 2.14 |
|  | ACC | 0.59 | 26 | 0.70 | 0.73 | 38 | 1.01 | 0.52 | 24 | 0.66 | 0.59 | 26 | 0.70 | 0.47 | 21 | 0.57 | 0.88 | 36 | 0.98 | 0.68 | 32 | 0.87 |
|  | ACA | 1.73 | 76 | 2.06 | 1.52 | 79 | 2.10 | 1.51 | 69 | 1.89 | 1.61 | 71 | 1.93 | 1.67 | 75 | 2.03 | 1.37 | 56 | 1.53 | 1.54 | 73 | 1.97 |
|  | ACG | 0.09 | 4 | 0.11 | 0.33 | 17 | 0.45 | 0.15 | 7 | 0.19 | 0.09 | 4 | 0.11 | 0.11 | 5 | 0.14 | 0.37 | 15 | 0.41 | 0.11 | 5 | 0.14 |
| Ala | GCU | 2.05 | 106 | 2.87 | 1.66 | 72 | 1.92 | 1.86 | 103 | 2.82 | 2.08 | 108 | 2.93 | 1.92 | 101 | 2.74 | 1.77 | 105 | 2.86 | 2.02 | 109 | 2.95 |
|  | GCC | 0.77 | 40 | 1.08 | 0.90 | 39 | 1.04 | 0.89 | 49 | 1.34 | 0.67 | 35 | 0.95 | 0.86 | 45 | 1.22 | 1.08 | 64 | 1.74 | 0.72 | 39 | 1.05 |
|  | GCA | 1.10 | 57 | 1.54 | 1.13 | 49 | 1.30 | 0.98 | 54 | 1.48 | 1.13 | 59 | 1.60 | 0.95 | 50 | 1.36 | 0.81 | 48 | 1.31 | 1.11 | 60 | 1.62 |
|  | GCG | 0.08 | 4 | 0.11 | 0.30 | 13 | 0.35 | 0.27 | 15 | 0.41 | 0.12 | 6 | 0.16 | 0.27 | 14 | 0.38 | 0.34 | 20 | 0.55 | 0.15 | 8 | 0.22 |
| Tyr | UAU | 1.46 | 100 | 2.71 | 1.11 | 83 | 2.21 | 1.31 | 88 | 2.41 | 1.49 | 102 | 2.77 | 1.32 | 89 | 2.41 | 1.35 | 75 | 2.04 | 1.42 | 97 | 2.62 |
|  | UAC | 0.54 | 37 | 1.00 | 0.89 | 66 | 1.76 | 0.69 | 46 | 1.26 | 0.51 | 35 | 0.95 | 0.68 | 46 | 1.25 | 0.65 | 36 | 0.98 | 0.58 | 40 | 1.08 |
| His | CAU | 1.37 | 54 | 1.46 | 0.99 | 36 | 0.96 | 1.21 | 47 | 1.29 | 1.30 | 46 | 1.25 | 1.32 | 49 | 1.33 | 0.94 | 33 | 0.90 | 1.28 | 46 | 1.24 |
|  | CAC | 0.63 | 25 | 0.68 | 1.01 | 37 | 0.98 | 0.79 | 31 | 0.85 | 0.70 | 25 | 0.68 | 0.68 | 25 | 0.68 | 1.06 | 37 | 1.01 | 0.72 | 26 | 0.70 |
| Gln | CAA | 1.70 | 51 | 1.38 | 1.19 | 34 | 0.90 | 1.20 | 42 | 1.15 | 1.42 | 46 | 1.25 | 1.44 | 46 | 1.25 | 1.63 | 53 | 1.44 | 1.31 | 42 | 1.14 |
|  | CAG | 0.30 | 9 | 0.24 | 0.81 | 23 | 0.61 | 0.80 | 28 | 0.77 | 0.58 | 19 | 0.52 | 0.56 | 18 | 0.49 | 0.37 | 12 | 0.33 | 0.69 | 22 | 0.60 |
| Asn | AAU | 1.31 | 81 | 2.19 | 1.19 | 80 | 2.13 | 1.25 | 74 | 2.03 | 1.40 | 96 | 2.60 | 1.37 | 90 | 2.44 | 1.15 | 61 | 1.66 | 1.39 | 91 | 2.46 |
|  | AAC | 0.69 | 43 | 1.16 | 0.81 | 55 | 1.46 | 0.75 | 44 | 1.20 | 0.60 | 41 | 1.11 | 0.63 | 41 | 1.11 | 0.85 | 45 | 1.23 | 0.61 | 40 | 1.08 |
| Lys | AAA | 1.63 | 79 | 2.14 | 1.60 | 100 | 2.66 | 1.60 | 71 | 1.94 | 1.47 | 69 | 1.87 | 1.47 | 69 | 1.87 | 1.25 | 57 | 1.55 | 1.60 | 76 | 2.06 |
|  | AAG | 0.37 | 18 | 0.49 | 0.40 | 25 | 0.67 | 0.40 | 18 | 0.49 | 0.53 | 25 | 0.68 | 0.53 | 25 | 0.68 | 0.75 | 34 | 0.93 | 0.40 | 19 | 0.51 |
| Asp | GAU | 1.24 | 44 | 1.19 | 1.17 | 42 | 1.12 | 1.13 | 44 | 1.20 | 1.41 | 52 | 1.41 | 1.30 | 48 | 1.30 | 1.32 | 45 | 1.23 | 1.39 | 52 | 1.41 |
|  | GAC | 0.76 | 27 | 0.73 | 0.83 | 30 | 0.80 | 0.87 | 34 | 0.93 | 0.59 | 22 | 0.60 | 0.70 | 26 | 0.71 | 0.68 | 23 | 0.63 | 0.61 | 23 | 0.62 |
| Glu | GAA | 1.35 | 58 | 1.57 | 1.01 | 37 | 0.98 | 1.31 | 53 | 1.45 | 1.24 | 51 | 1.38 | 1.33 | 55 | 1.49 | 1.09 | 44 | 1.20 | 1.40 | 58 | 1.57 |
|  | GAG | 0.65 | 28 | 0.76 | 0.99 | 36 | 0.96 | 0.69 | 28 | 0.77 | 0.76 | 31 | 0.84 | 0.67 | 28 | 0.76 | 0.91 | 37 | 1.01 | 0.60 | 25 | 0.68 |
| Cys | UGU | 1.62 | 30 | 0.81 | 1.59 | 35 | 0.93 | 1.25 | 25 | 0.68 | 1.49 | 26 | 0.70 | 1.60 | 28 | 0.76 | 1.33 | 24 | 0.65 | 1.56 | 28 | 0.76 |
|  | UGC | 0.38 | 7 | 0.19 | 0.41 | 9 | 0.24 | 0.75 | 15 | 0.41 | 0.51 | 9 | 0.24 | 0.40 | 7 | 0.19 | 0.67 | 12 | 0.33 | 0.44 | 8 | 0.22 |
| Trp | UGA | 1.53 | 74 | 2.00 | 1.29 | 65 | 1.73 | 1.28 | 59 | 1.62 | 1.30 | 60 | 1.63 | 1.40 | 65 | 1.76 | 1.10 | 55 | 1.50 | 1.61 | 75 | 2.03 |
|  | UGG | 0.47 | 23 | 0.62 | 0.71 | 36 | 0.96 | 0.72 | 33 | 0.90 | 0.70 | 32 | 0.87 | 0.60 | 28 | 0.76 | 0.90 | 45 | 1.23 | 0.39 | 18 | 0.49 |
| Arg | CGU | 1.36 | 17 | 0.46 | 1.11 | 15 | 0.40 | 1.46 | 19 | 0.52 | 1.54 | 20 | 0.54 | 1.23 | 16 | 0.43 | 0.64 | 9 | 0.25 | 1.23 | 16 | 0.43 |
|  | CGC | 0.32 | 4 | 0.11 | 0.44 | 6 | 0.16 | 0.54 | 7 | 0.19 | 0.38 | 5 | 0.14 | 0.69 | 9 | 0.24 | 1.00 | 14 | 0.38 | 0.54 | 7 | 0.19 |
|  | CGA | 1.76 | 22 | 0.60 | 1.19 | 16 | 0.43 | 1.08 | 14 | 0.38 | 1.38 | 18 | 0.49 | 1.31 | 17 | 0.46 | 1.29 | 18 | 0.49 | 1.85 | 24 | 0.65 |
|  | CGG | 0.56 | 7 | 0.19 | 1.26 | 17 | 0.45 | 0.92 | 12 | 0.33 | 0.69 | 9 | 0.24 | 0.77 | 10 | 0.27 | 1.07 | 15 | 0.41 | 0.38 | 5 | 0.14 |
| Ser1 | AGU | 1.10 | 59 | 1.60 | 1.18 | 67 | 1.78 | 1.08 | 55 | 1.51 | 1.09 | 57 | 1.55 | 1.10 | 58 | 1.57 | 0.75 | 41 | 1.12 | 0.96 | 49 | 1.33 |
|  | AGC | 0.21 | 11 | 0.30 | 0.39 | 22 | 0.59 | 0.41 | 21 | 0.58 | 0.23 | 12 | 0.33 | 0.38 | 20 | 0.54 | 0.46 | 25 | 0.68 | 0.21 | 11 | 0.30 |
|  | AGA | 1.86 | 100 | 2.71 | 1.47 | 84 | 2.23 | 1.97 | 100 | 2.74 | 2.03 | 106 | 2.87 | 1.87 | 99 | 2.69 | 1.59 | 87 | 2.37 | 1.85 | 95 | 2.57 |
|  | AGG | 0.75 | 40 | 1.08 | 0.79 | 45 | 1.20 | 0.69 | 35 | 0.96 | 0.57 | 30 | 0.81 | 0.62 | 33 | 0.90 | 1.03 | 56 | 1.53 | 0.90 | 46 | 1.24 |
| Gly | GGU | 1.02 | 58 | 1.57 | 1.11 | 61 | 1.62 | 1.12 | 67 | 1.83 | 1.06 | 62 | 1.68 | 1.08 | 64 | 1.74 | 0.86 | 54 | 1.47 | 1.07 | 63 | 1.70 |
|  | GGC | 0.53 | 30 | 0.81 | 0.82 | 45 | 1.20 | 0.43 | 26 | 0.71 | 0.43 | 25 | 0.68 | 0.54 | 32 | 0.87 | 0.83 | 52 | 1.42 | 0.39 | 23 | 0.62 |
|  | GGA | 1.44 | 82 | 2.22 | 1.11 | 61 | 1.62 | 1.17 | 70 | 1.92 | 1.57 | 92 | 2.49 | 1.29 | 76 | 2.06 | 0.91 | 57 | 1.55 | 1.47 | 87 | 2.35 |
|  | GGG | 1.00 | 57 | 1.54 | 0.95 | 52 | 1.38 | 1.28 | 77 | 2.11 | 0.94 | 55 | 1.49 | 1.08 | 64 | 1.74 | 1.39 | 87 | 2.37 | 1.07 | 63 | 1.70 |

**Supplementary Table S6.** Summary of amino acid composition in PCGs of mitochondrial genomes of all crangonyctid amphipods. Sign “#” indicates total number of certain amino acid in protein-coding sequences of every species; “%” indicates percent of certain amino acid in total coding sequence in every species

| **AA** | **Stygobromus tenuis potomacus** | | **Stygobromus indentatus** | | **Stygobromus allegheniensis** | | **Bactrurus brachycaudus** | | **Crangonyx forbesi** | | **Stygobromus pizzinii** | | **Stygobromus tenuis potomacus** | |
| --- | --- | --- | --- | --- | --- | --- | --- | --- | --- | --- | --- | --- | --- | --- |
|  | **#** | **%** | **#** | **%** | **#** | **%** | **#** | **%** | **#** | **%** | **#** | **%** | **#** | **%** |
| Phe | 282 | 7.63 | 273 | 7.40 | 289 | 7.91 | 281 | 7.66 | 290 | 7.71 | 286 | 7.76 | 282 | 7.65 |
| Leu2 | 370 | 10.01 | 334 | 9.05 | 349 | 9.56 | 281 | 7.66 | 333 | 8.86 | 355 | 9.63 | 363 | 9.84 |
| Leu1 | 249 | 6.74 | 259 | 7.02 | 258 | 7.06 | 310 | 8.45 | 287 | 7.64 | 247 | 6.70 | 243 | 6.59 |
| Ile | 301 | 8.14 | 323 | 8.75 | 259 | 7.09 | 271 | 7.39 | 251 | 6.68 | 304 | 8.25 | 296 | 8.03 |
| Met | 198 | 5.36 | 230 | 6.23 | 182 | 4.98 | 224 | 6.11 | 228 | 6.07 | 204 | 5.53 | 206 | 5.59 |
| Val | 258 | 6.98 | 248 | 6.72 | 287 | 7.86 | 282 | 7.69 | 279 | 7.42 | 258 | 7.00 | 274 | 7.43 |
| Ser2 | 209 | 5.65 | 219 | 5.93 | 195 | 5.34 | 228 | 6.22 | 238 | 6.33 | 213 | 5.78 | 213 | 5.78 |
| Pro | 150 | 4.06 | 147 | 3.98 | 146 | 4.00 | 148 | 4.03 | 152 | 4.04 | 149 | 4.04 | 149 | 4.04 |
| Thr | 189 | 5.11 | 176 | 4.77 | 183 | 5.01 | 163 | 4.44 | 208 | 5.53 | 180 | 4.88 | 176 | 4.77 |
| Ala | 216 | 5.84 | 207 | 5.61 | 221 | 6.05 | 237 | 6.46 | 173 | 4.60 | 210 | 5.70 | 208 | 5.64 |
| Tyr | 137 | 3.71 | 137 | 3.71 | 134 | 3.67 | 111 | 3.03 | 149 | 3.96 | 135 | 3.66 | 137 | 3.71 |
| His | 72 | 1.95 | 79 | 2.14 | 78 | 2.14 | 70 | 1.91 | 73 | 1.94 | 74 | 2.01 | 71 | 1.93 |
| Gln | 64 | 1.73 | 60 | 1.63 | 70 | 1.92 | 65 | 1.77 | 57 | 1.52 | 64 | 1.74 | 65 | 1.76 |
| Asn | 131 | 3.54 | 124 | 3.36 | 118 | 3.23 | 106 | 2.89 | 135 | 3.59 | 131 | 3.55 | 137 | 3.71 |
| Lys | 95 | 2.57 | 97 | 2.63 | 89 | 2.44 | 91 | 2.48 | 125 | 3.33 | 94 | 2.55 | 94 | 2.55 |
| Asp | 75 | 2.03 | 71 | 1.92 | 78 | 2.14 | 68 | 1.85 | 72 | 1.92 | 74 | 2.01 | 74 | 2.01 |
| Glu | 83 | 2.25 | 86 | 2.33 | 81 | 2.22 | 81 | 2.21 | 73 | 1.94 | 83 | 2.25 | 82 | 2.22 |
| Cys | 36 | 0.97 | 37 | 1.00 | 40 | 1.10 | 36 | 0.98 | 44 | 1.17 | 35 | 0.95 | 35 | 0.95 |
| Trp | 93 | 2.52 | 97 | 2.63 | 92 | 2.52 | 100 | 2.73 | 101 | 2.69 | 93 | 2.52 | 92 | 2.49 |
| Arg | 52 | 1.41 | 50 | 1.35 | 52 | 1.42 | 56 | 1.53 | 54 | 1.44 | 52 | 1.41 | 52 | 1.41 |
| Ser1 | 201 | 5.44 | 210 | 5.69 | 211 | 5.78 | 209 | 5.70 | 218 | 5.80 | 210 | 5.70 | 205 | 5.56 |
| Gly | 236 | 6.38 | 227 | 6.15 | 240 | 6.57 | 250 | 6.82 | 219 | 5.83 | 236 | 6.40 | 234 | 6.34 |

**Supplementary Table S7.** Nucleotide composition statistics for ribosomal RNA genes in mitochondrial genomes of all crangonyctid amphipods.

| **Species** | **Gene** | **Size (bp)** | **AT %** | **AT-skew** | **GC-skew** |
| --- | --- | --- | --- | --- | --- |
| *Bactrurus brachycaudus* | *rrnL-* | 1034 | 67.8 | -0.030 | 0.381 |
|  | *rrnS-* | 671 | 71.5 | -0.029 | 0.361 |
| *Crangonyx forbesi* | *rrnL-* | 1090 | 72.7 | -0.064 | 0.259 |
|  | *rrnS-* | 695 | 73.6 | -0.084 | 0.359 |
| *Stygobromus allegheniensis* | *rrnL-* | 1034 | 70.4 | -0.115 | 0.412 |
|  | *rrnS-* | 688 | 74.0 | -0.108 | 0.352 |
| *Stygobromus indentatus* | *rrnL-* | 1035 | 72.7 | -0.089 | 0.383 |
|  | *rrnS-* | 669 | 77.2 | -0.070 | 0.307 |
| *Stygobromus pizzinii* | *rrnL-* | 1037 | 72.3 | -0.093 | 0.415 |
|  | *rrnS-* | 678 | 74.6 | -0.075 | 0.337 |
| *Stygobromus tenuis potomacus* | *rrnL-* | 1036 | 72.8 | -0.074 | 0.426 |
|  | *rrnS-* | 679 | 76.0 | -0.109 | 0.370 |
| *Stygobromus tenuis potomacus* | *rrnL-* | 1034 | 72.3 | -0.099 | 0.420 |
|  | *rrnS-* | 683 | 74.6 | -0.088 | 0.322 |

**Supplementary Table S8.** Results of selective pressure (ω ratio) analyses of mitochondrial PCGs with LRT P-value < 0.05 in subterranean and surface lineages of amphipods based on 2 vs. 1 ratio model.

| **Model** | **np** | **Ln L** | **Estimates of parameters** | | | **Model compared** | **LRT P-value** | **Omega for Foreground Branch** | **Gene** | **Species** |
| --- | --- | --- | --- | --- | --- | --- | --- | --- | --- | --- |
| Two ratio Model 2 | 70 | -24608.883248 | ω: | ω0=0.02037 | ω1=0.05379 | Model 0 vs. Two ratio Model 2 | 0.000131407 | ω1=0.05379 | *cox1* | *C. forbesi* |
| Model 0 | 69 | -24616.193996 | ω= | 0.02099 | |  |  |  |  |  |
| Two ratio Model 2 | 70 | -15283.383385 | ω: | ω0=0.05091 | ω1=0.18347 | Model 0 vs. Two ratio Model 2 | 0.037178941 | ω1=0.18347 | *atp6* | *P. daviui* |
| Model 0 | 69 | -15285.554483 | ω= | 0.05174 | |  |  |  |  |  |
| Two ratio Model 2 | 70 | -24611.896377 | ω: | ω0=0.02052 | ω1=0.04222 | Model 0 vs. Two ratio Model 2 | 0.003370432 | ω1=0.04222 | *cox1* |  |
| Model 0 | 69 | -24616.193996 | ω= | 0.02099 |  |  |  |  |  |  |
| Two ratio Model 2 | 70 | -19414.614776 | ω: | ω0=0.03225 | ω1=0.19046 | Model 0 vs. Two ratio Model 2 | 0.002904709 | ω1=0.19046 | *nad1* |  |
| Model 0 | 69 | -19419.047965 | ω= | 0.03239 | |  |  |  |  |  |
| Two ratio Model 2 | 70 | -24599.610909 | ω: | ω0=0.02012 | ω1=0.07488 | Model 0 vs. Two ratio Model 2 | 0.000000008 | ω1=0.07488 | *cox1* | *G. fasciatus* |
| Model 0 | 69 | -24616.193996 | ω= | 0.02099 | |  |  |  |  |  |
| Two ratio Model 2 | 70 | -12953.332620 | ω: | ω0=0.03009 | ω1=0.21013 | Model 0 vs. Two ratio Model 2 | 0.000072299 | ω1=0.21013 | *cox2* |  |
| Model 0 | 69 | -12961.207473 | ω= | 0.03033 | |  |  |  |  |  |
| Two ratio Model 2 | 70 | -16204.441186 | ω: | ω0=0.04169 | ω1=0.09747 | Model 0 vs. Two ratio Model 2 | 0.012180256 | ω1=0.09747 | *cox3* |  |
| Model 0 | 69 | -16207.583406 | ω= | 0.04274 | |  |  |  |  |  |
| Two ratio Model 2 | 70 | -29486.407012 | ω: | ω0=0.04262 | ω1=0.12215 | Model 0 vs. Two ratio Model 2 | 0.002680018 | ω1=0.12215 | *nad4* |  |
| Model 0 | 69 | -29490.913731 | ω= | 0.04296 | |  |  |  |  |  |
| Two ratio Model 2 | 70 | -16205.223658 | ω: | ω0=0.04182 | ω1=0.10051 | Model 0 vs. Two ratio Model 2 | 0.029822483 | ω1=0.10051 | *cox3* | *O. nanseni* |
| Model 0 | 69 | -16207.583406 | ω= | 0.04274 | |  |  |  |  |  |
| Two ratio Model 2 | 70 | -24619.041265 | ω: | ω0=0.01766 | ω1=0.04082 | Model 0 vs. Two ratio Model 2 | 0.017017792 | ω1=0.04082 | *cox1* | *G. fossarum* |
| Model 0 | 69 | -24616.193996 | ω= | 0.02099 |  |  |  |  |  |  |
| Two ratio Model 2 | 70 | -23400.542887 | ω: | ω0=0.03254 | ω1=0.08246 | Model 0 vs. Two ratio Model 2 | 0.004931290 | ω1=0.08246 | *cytb* |  |
| Model 0 | 69 | -23404.495120 | ω= | 0.03337 | |  |  |  |  |  |
| Two ratio Model 2 | 70 | -19414.218221 | ω: | ω0=0.03259 | ω1=0.13042 | Model 0 vs. Two ratio Model 2 | 0.001883761 | ω1=0.13042 | *nad1* |  |
| Model 0 | 69 | -19419.047965 | ω= | 0.03239 | |  |  |  |  |  |
| Two ratio Model 2 | 70 | -29487.580618 | ω: | ω0=0.04235 | ω1=0.09448 | Model 0 vs. Two ratio Model 2 | 0.009825704 | ω1=0.09448 | *nad4* |  |
| Model 0 | 69 | -29490.913731 | ω= | 0.04296 |  |  |  |  |  |  |
| Two ratio Model 2 | 70 | -6864.685694 | ω: | ω0=0.03733 | ω1=0.19416 | Model 0 vs. Two ratio Model 2 | 0.030276845 | ω1=0.19416 | *nad4l* |  |
| Model 0 | 69 | -6867.032446 | ω= | 0.03788 |  |  |  |  |  |  |
| Two ratio Model 2 | 70 | -24613.676186 | ω: | ω0=0.02143 | ω1=0.01086 | Model 0 vs. Two ratio Model 2 | 0.024831197 | ω1=0.01086 | *cox1* | *B. jaraguensis* |
| Model 0 | 69 | -24616.193996 | ω= | 0.02099 | |  |  |  |  |  |
| Two ratio Model 2 | 70 | -42645.830655 | ω: | ω0=0.06114 | ω1=0.02138 | Model 0 vs. Two ratio Model 2 | 0.035443809 | ω1=0.02138 | *nad5* |  |
| Model 0 | 69 | -42648.042488 | ω= | 0.06077 | |  |  |  |  |  |
| Two ratio Model 2 | 70 | -24610.442091 | ω: | ω0=0.02042 | ω1=0.04738 | Model 0 vs. Two ratio Model 2 | 0.000694537 | ω1=0.04738 | *cox1* | *B. brachycaudus* |
| Model 0 | 69 | -24616.193996 | ω= | 0.02099 |  |  |  |  |  |  |
| Two ratio Model 2 | 70 | -16205.427054 | ω: | ω0=0.04211 | ω1=0.09064 | Model 0 vs. Two ratio Model 2 | 0.037828788 | ω1=0.09064 | *cox3* |  |
| Model 0 | 69 | -16207.583406 | ω= | 0.04274 |  |  |  |  |  |  |
| Two ratio Model 2 | 70 | -19416.309168 | ω: | ω0=0.03286 | ω1=0.00207 | Model 0 vs. Two ratio Model 2 | 0.019261754 | ω1=0.00207 | *nad1* |  |
| Model 0 | 69 | -19419.047965 | ω= | 0.03239 |  |  |  |  |  |  |
| Two ratio Model 2 | 70 | -24607.788487 | ω: | ω0=0.02132 | ω1=0.00010 | Model 0 vs. Two ratio Model 2 | 0.000041293 | ω1=0.00010 | *cox1* | *S. tenuis_MN* |
| Model 0 | 69 | -24616.193996 | ω= | 0.02099 |  |  |  |  |  |  |
| Two ratio Model 2 | 70 | -12967.565600 | ω: | ω0=0.02987 | ω1=2.22100 | Model 0 vs. Two ratio Model 2 | 0.000362491 | ω1=2.22100 | *cox2* |  |
| Model 0 | 69 | -12961.207473 | ω= | 0.03033 | |  |  |  |  |  |
| Two ratio Model 2 | 70 | -19417.045819 | ω: | ω0=0.03302 | ω1=0.00531 | Model 0 vs. Two ratio Model 2 | 0.045384555 | ω1=0.00531 | *nad1* |  |
| Model 0 | 69 | -19419.047965 | ω= | 0.03239 |  |  |  |  |  |  |
| Two ratio Model 2 | 70 | -6864.477274 | ω: | ω0=0.03988 | ω1=0.00010 | Model 0 vs. Two ratio Model 2 | 0.023783604 | ω1=0.00010 | *nad4l* |  |
| Model 0 | 69 | -6867.032446 | ω= | 0.03788 | |  |  |  |  |  |
| Two ratio Model 2 | 70 | -42645.097762 | ω: | ω0=0.06190 | ω1=0.02375 | Model 0 vs. Two ratio Model 2 | 0.015231840 | ω1=0.02375 | *nad5* |  |
| Model 0 | 69 | -42648.042488 | ω= | 0.06077 | |  |  |  |  |  |
| Two ratio Model 2 | 70 | -24643.013443 | ω: | ω0=0.02059 | ω1=37.72058 | Model 0 vs. Two ratio Model 2 | 0.000000000 | ω1=37.72058 | *cox1* | *S. tenuis_KU* |
| Model 0 | 69 | -24616.193996 | ω= | 0.02099 | |  |  |  |  |  |
| Two ratio Model 2 | 70 | -16204.089657 | ω: | ω0=0.04368 | ω1=0.01396 | Model 0 vs. Two ratio Model 2 | 0.008208101 | ω1=0.01396 | *cox3* |  |
| Model 0 | 69 | -16207.583406 | ω= | 0.04274 | |  |  |  |  |  |
| Two ratio Model 2 | 70 | -42643.975206 | ω: | ω0=0.06250 | ω1=0.02993 | Model 0 vs. Two ratio Model 2 | 0.004342929 | ω1=0.02993 | *nad5* |  |
| Model 0 | 69 | -42648.042488 | ω= | 0.06077 | |  |  |  |  |  |
| Two ratio Model 2 | 70 | -11161.910962 | ω: | ω0=0.04608 | ω1=0.01015 | Model 0 vs. Two ratio Model 2 | 0.045732562 | ω1=0.01015 | *nad6* |  |
| Model 0 | 69 | -11163.906671 | ω= | 0.04373 | |  |  |  |  |  |
| Two ratio Model 2 | 70 | -24609.090352 | ω: | ω0=0.02152 | ω1=0.00488 | Model 0 vs. Two ratio Model 2 | 0.000163735 | ω1=0.00488 | *cox1* | *S. allegheniensis* |
| Model 0 | 69 | -24616.193996 | ω= | 0.02099 | |  |  |  |  |  |
| Two ratio Model 2 | 70 | -16205.109405 | ω: | ω0=0.04350 | ω1=0.01645 | Model 0 vs. Two ratio Model 2 | 0.026120839 | ω1=0.01645 | *cox3* |  |
| Model 0 | 69 | -16207.583406 | ω= | 0.04274 | |  |  |  |  |  |
| Two ratio Model 2 | 70 | -24613.689587 | ω: | ω0=0.02117 | ω1=0.00359 | Model 0 vs. Two ratio Model 2 | 0.025218520 | ω1=0.00359 | *cox1* | *S. pizzinii* |
| Model 0 | 69 | -24616.193996 | ω= | 0.02099 | |  |  |  |  |  |
| Two ratio Model 2 | 70 | -12958.490043 | ω: | ω0=0.03112 | ω1=0.00477 | Model 0 vs. Two ratio Model 2 | 0.019738673 | ω1=0.00477 | *cox2* |  |
| Model 0 | 69 | -12961.207473 | ω= | 0.03033 | |  |  |  |  |  |
| Two ratio Model 2 | 70 | -23400.497996 | ω: | ω0=0.03415 | ω1=0.00760 | Model 0 vs. Two ratio Model 2 | 0.004692619 | ω1=0.00760 | *cytb* |  |
| Model 0 | 69 | -23404.495120 | ω= | 0.03337 | |  |  |  |  |  |
| Two ratio Model 2 | 70 | -29487.040979 | ω: | ω0=0.04390 | ω1=0.01007 | Model 0 vs. Two ratio Model 2 | 0.005384643 | ω1=0.01007 | *nad4* |  |
| Model 0 | 69 | -29490.913731 | ω= | 0.04296 | |  |  |  |  |  |
| Two ratio Model 2 | 70 | -24606.402409 | ω: | ω0=0.02171 | ω1=0.00333 | Model 0 vs. Two ratio Model 2 | 0.000009631 | ω1=0.00333 | *cox1* | *S. indentatus* |
| Model 0 | 69 | -24616.193996 | ω= | 0.02099 | |  |  |  |  |  |
| Two ratio Model 2 | 70 | -19831.073084 | ω: | ω0=0.04595 | ω1=0.00102 | Model 0 vs. Two ratio Model 2 | 0.000440467 | ω1=0.00102 | *nad2* |  |
| Model 0 | 69 | -19837.249185 | ω= | 0.04445 | |  |  |  |  |  |

**Supplementary Table S9.** Evidence of positive selection on the mitochondrial PCGs with LRT P-value < 0.05 and positively selected site (BEB: P≥95%) in subterranean and surface dwelling lineages of amphipods based on branch-site models.

| **Species** | **Gene** | **Model** | **np** | **Ln L** | **Estimates of parameters** | | | | | **Model compared** | **LRT P-value** | **Positive sites (BEB: P≥95%)** |
| --- | --- | --- | --- | --- | --- | --- | --- | --- | --- | --- | --- | --- |
| *P. kessleri* | *atp8* | Model A | 72 | -3843.85 | Site class | 0 | 1 | 2a | 2b | Model A vs.Model A null | 0.0007 | 4 S 1.000**,5 N 0.978*,6 W 1.000**,8 F 1.000**,14 L 0.969*,16 I 0.981*,20 M 0.976*,21 N 0.952*,24 L 0.999**,28 S 0.988*,33 L 0.951*,34 N 0.958*,35 N 0.993**,37 A 0.986* |
|  |  |  |  |  | f | 0.23149 | 0.32042 | 0.18795 | 0.26014 |  |  |  |
|  |  |  |  |  | ω0 | 0.12034 | 1.00000 | 0.12034 | 1.00000 |  |  |  |
|  |  |  |  |  | ω1 | 0.12034 | 1.00000 | 999.00000 | 999.00000 |  |  |  |
|  |  | Model A null | 71 | -3849.58 | 1 |  |  |  |  |  |  | Not Allowed |
|  | *nad5* | Model A | 72 | -42215.62 | Site class | 0 | 1 | 2a | 2b | Model A vs.Model A null | 0.0468 | 12 G 0.992**,34 W 0.993**,60 T 0.964*,91 M 0.966*,358 L 0.971*,457 K 0.964*,477 S 0.979*,511 S 0.960* |
|  |  |  |  |  | f | 0.76401 | 0.11614 | 0.10404 | 0.01581 |  |  |  |
|  |  |  |  |  | ω0 | 0.06917 | 1.00000 | 0.06917 | 1.00000 |  |  |  |
|  |  |  |  |  | ω1 | 0.06917 | 1.00000 | 2.17571 | 2.17571 |  |  |  |
|  |  | Model A null | 71 | -42217.60 | 1 |  |  |  |  |  |  | Not Allowed |
| *G. antarctica* | *nad5* | Model A | 72 | -42207.04 | Site class | 0 | 1 | 2a | 2b | Model A vs.Model A null | 0.0045 | 35 G 0.996**,40 N 0.971*,46 F 0.994**,47 S 0.957*,59 S 0.985*,131 S 0.986*,171 S 0.953*,192 S 0.989*,239 I 0.965*,283 E 0.993**,381 V 0.980*,389 M 0.993**,429 W 0.953*,472 G 0.987*,473 L 0.991**,477 S 0.976*,488 K 0.962* |
|  |  |  |  |  | f | 0.69413 | 0.11323 | 0.16562 | 0.02702 |  |  |  |
|  |  |  |  |  | ω0 | 0.07063 | 1.00000 | 0.07063 | 1.00000 |  |  |  |
|  |  |  |  |  | ω1 | 0.07063 | 1.00000 | 42.86420 | 42.86420 |  |  |  |
|  |  | Model A null | 71 | -42211.07 | 1 |  |  |  |  |  |  | Not Allowed |
|  | *nad6* | Model A | 72 | -11115.29 | Site class | 0 | 1 | 2a | 2b | Model A vs.Model A null | 0.0019 | 51 G 0.989*,86 N 0.982*,100 S 0.964*,108 L 0.954*,128 G 0.974* |
|  |  |  |  |  | f | 0.60982 | 0.15741 | 0.18502 | 0.04776 |  |  |  |
|  |  |  |  |  | ω0 | 0.07302 | 1.00000 | 0.07302 | 1.00000 |  |  |  |
|  |  |  |  |  | ω1 | 0.07302 | 1.00000 | 1.50615 | 1.50615 |  |  |  |
|  |  | Model A null | 71 | -11110.47 | 1 |  |  |  |  |  |  | Not Allowed |
| *P. japonica* | *nad2* | Model A | 72 | -19775.06 | Site class | 0 | 1 | 2a | 2b | Model A vs.Model A null | 0.0172 | 116 D 0.979*, 232 S 0.970* |
|  |  |  |  |  | f | 0.92964 | 0.04229 | 0.02685 | 0.00122 |  |  |  |
|  |  |  |  |  | ω0 | 0.04908 | 1.00000 | 0.04908 | 1.00000 |  |  |  |
|  |  |  |  |  | ω1 | 0.04908 | 1.00000 | 855.94976 | 855.94976 |  |  |  |
|  |  | Model A null | 71 | -19777.90 | 1 |  |  |  |  |  |  | Not Allowed |
|  | *nad6* | Model A | 72 | -11114.94 | Site class | 0 | 1 | 2a | 2b | Model A vs.Model A null | 0.0420 | 86 N 0.982* |
|  |  |  |  |  | f | 0.80724 | 0.14323 | 0.04207 | 0.00746 |  |  |  |
|  |  |  |  |  | ω0 | 0.06706 | 1.00000 | 0.06706 | 1.00000 |  |  |  |
|  |  |  |  |  | ω1 | 0.06706 | 1.00000 | 998.99536 | 998.99536 |  |  |  |
|  |  | Model A null | 71 | -11117.00 | 1 |  |  |  |  |  |  | Not Allowed |
| *C. mutica* | *nad1* | Model A | 72 | -19367.63 | Site class | 0 | 1 | 2a | 2b | Model A vs.Model A null | 0.0007 | 163 I 0.997**,243 S 0.984*,249 V 0.989* |
|  |  |  |  |  | f | 0.94079 | 0.02466 | 0.03367 | 0.00088 |  |  |  |
|  |  |  |  |  | ω0 | 0.03489 | 1.00000 | 0.03489 | 1.00000 |  |  |  |
|  |  |  |  |  | ω1 | 0.03489 | 1.00000 | 14.28437 | 14.28437 |  |  |  |
|  |  | Model A null | 71 | -19373.41 | 1 |  |  |  |  |  |  | Not Allowed |
|  | *nad5* | Model A | 72 | -42225.68 | Site class | 0 | 1 | 2a | 2b | Model A vs.Model A null | 0.0030 | 280 G 0.975*,326 G 0.975*,400 S 0.989* |
|  |  |  |  |  | f | 0.79382 | 0.13139 | 0.06417 | 0.01062 |  |  |  |
|  |  |  |  |  | ω0 | 0.07070 | 1.00000 | 0.07070 | 1.00000 |  |  |  |
|  |  |  |  |  | ω1 | 0.07070 | 1.00000 | 9.66169 | 9.66169 |  |  |  |
|  |  | Model A null | 71 | -42230.09 | 1 |  |  |  |  |  |  | Not Allowed |
|  | *cox2* | Model A | 72 | -12930.83 | Site class | 0 | 1 | 2a | 2b | Model A vs.Model A null | 0.0115 | 142 K 0.986* |
|  |  |  |  |  | f | 0.97663 | 0.00901 | 0.01423 | 0.00013 |  |  |  |
|  |  |  |  |  | ω0 | 0.03012 | 1.00000 | 0.03012 | 1.00000 |  |  |  |
|  |  |  |  |  | ω1 | 0.03012 | 1.00000 | 268.52413 | 268.52413 |  |  |  |
|  |  | Model A null | 71 | -12934.02 | 1 |  |  |  |  |  |  | Not Allowed |
| *G. fossarum* | *nad3* | Model A | 72 | -7315.91 | Site class | 0 | 1 | 2a | 2b | Model A vs.Model A null | 0.0439 | 6 F 0.991**,17 L 0.999**,22 H 0.979*,23 S 0.999**,25 P 0.969*,26 S 1.000**,76 P 0.978*,82 T 0.972*,84 L 0.998**,91 V 0.974*,93 L 0.966*,94 I 0.999** |
|  |  |  |  |  | f | 0.59041 | 0.08730 | 0.28077 | 0.04152 |  |  |  |
|  |  |  |  |  | ω0 | 0.03895 | 1.00000 | 0.03895 | 1.00000 |  |  |  |
|  |  |  |  |  | ω1 | 0.03895 | 1.00000 | 11.02113 | 11.02113 |  |  |  |
|  |  | Model A null | 71 | -7317.94 | 1 |  |  |  |  |  |  | Not Allowed |
| *O. nanseni* | *atp6* | Model A | 72 | -15167.07 | Site class | 0 | 1 | 2a | 2b | Model A vs.Model A null | 0.0478 | 69 M 0.986*, 146 N 0.981*, 172 S 0.974*, 186 A 0.969*, 188 G 0.981*, 189 L 0.996** |
|  |  |  |  |  | f | 0.76666 | 0.07611 | 0.14304 | 0.01420 |  |  |  |
|  |  |  |  |  | ω0 | 0.05002 | 1.00000 | 0.05002 | 1.00000 |  |  |  |
|  |  |  |  |  | ω1 | 0.05002 | 1.00000 | 18.13180 | 18.13180 |  |  |  |
|  |  | Model A null | 71 | -15169.03 | 1 |  |  |  |  |  |  | Not Allowed |
|  | *nad5* | Model A | 72 | -42207.40 | Site class | 0 | 1 | 2a | 2b | Model A vs.Model A null | 0.0004 | 3 S 0.991**, 6 A 0.988*, 73 S 0.985*, 98 F 0.972*, 130 S 0.968*, 162 W 0.961*, 179 I 0.960*, 242 M 0.955*, 325 W 0.976* , 341 C 0.995**, 367 L 0.990**, 373 V 0.953*, 377 G 0.974*, 427 M 0.987*, 465 N 0.989*, 479 W 0.977*, 514 L 0.969*, 515 Q 0.989*, 518 Q 0.999**, 519 S 0.985*, 523 S 0.959* |
|  |  |  |  |  | f | 0.72687 | 0.12217 | 0.12924 | 0.02172 |  |  |  |
|  |  |  |  |  | ω0 | 0.07034 | 1.00000 | 0.07034 | 1.00000 |  |  |  |
|  |  |  |  |  | ω1 | 0.07034 | 1.00000 | 62.60443 | 62.60443 |  |  |  |
|  |  | Model A null | 71 | -42213.74 | 1 |  |  |  |  |  |  | Not Allowed |
| *G. fasciatus* | *atp8* | Model A | 72 | -4021.06 | Site class | 0 | 1 | 2a | 2b | Model A vs.Model A null | 0.0000 | 7 S 0.999**, 12 F 0.976* |
|  |  |  |  |  | f | 0.25262 | 0.42481 | 0.12029 | 0.20228 |  |  |  |
|  |  |  |  |  | ω0 | 0.09609 | 1.00000 | 0.09609 | 1.00000 |  |  |  |
|  |  |  |  |  | ω1 | 0.09609 | 1.00000 | 327.33424 | 327.33424 |  |  |  |
|  |  | Model A null | 71 | -3851.18 | 1 |  |  |  |  |  |  | Not Allowed |
|  | *nad2* | Model A | 72 | -19783.73 | Site class | 0 | 1 | 2a | 2b | Model A vs.Model A null | 0.0000 | 117 M 0.960*, 191 K 0.967* |
|  |  |  |  |  | f | 0.80648 | 0.03554 | 0.15131 | 0.00667 |  |  |  |
|  |  |  |  |  | ω0 | 0.04922 | 1.00000 | 0.04922 | 1.00000 |  |  |  |
|  |  |  |  |  | ω1 | 0.04922 | 1.00000 | 1.00000 | 1.00000 |  |  |  |
|  |  | Model A null | 71 | -19775.46 | 1 |  |  |  |  |  |  | Not Allowed |
|  | *nad5* | Model A | 72 | -42209.09 | Site class | 0 | 1 | 2a | 2b | Model A vs.Model A null | 0.0001 | 17 V 0.993**, 27 N 0.977*, 72 S 0.978*, 159 S 0.972*, 161 G 0.994**, 183 V 0.961*, 242 M 0.976*, 243 M 0.978*, 313 S 0.966*, 323 G 0.985*, 368 C 0.960*, 376 S 0.962*, 388 S 0.996**, 389 M 0.852, 391 V 0.984*, 474 A 0.997**, 514 L 0.976* |
|  |  |  |  |  | f | 0.75033 | 0.12088 | 0.11092 | 0.01787 |  |  |  |
|  |  |  |  |  | ω0 | 0.06986 | 1.00000 | 0.06986 | 1.00000 |  |  |  |
|  |  |  |  |  | ω1 | 0.06986 | 1.00000 | 94.94243 | 94.94243 |  |  |  |
|  |  | Model A null | 71 | -42216.31 | 1 |  |  |  |  |  |  | Not Allowed |
| *C. forbesi* | *nad5* | Model A | 72 | -42180.92 | Site class | 0 | 1 | 2a | 2b | Model A vs.Model A null | 0.0000 | 2 F 0.982*, 20 S 0.986*, 23 V 0.994**, 40 N 0.978*, 48 L 0.979*, 56 V 0.970*, 78 G 0.991**, 82 I 0.993**, 88 I 0.958*, 121 A 0.997**, 131 S 0.992**, 132 Q 0.999**, 152 S 0.981*, 157 S 0.979*, 159 S 0.989*, 165 V 0.965*, 178 I 0.962*, 181 A 0.990*, 192 S 0.998**, 200 A 0.997**, 250 L 0.989*, 253 N 0.980*, 254 Y 0.991**, 271 G 0.995**, 278 S 0.989*, 283 E 0.989*, 323 G 0.978*, 333 F 0.966*, 336 C 0.991**, 337 N 0.999**, 344 P 0.957*, 347 S 0.996**, 354 L 0.983*, 355 V 0.956*, 359 M 0.725, 360 L 0.956*, 361 S 0.996**, 373 V 0.989*, 375 S 0.988*, 384 V 0.989*, 411 G 0.999**, 412 G 0.956*, 417 F 0.984*, 444 S 0.981*, 448 L 0.984*, 465 N 0.986*, 472 G 0.996**, 473 L 0.994**, 474 A 0.998**, 495 L 0.997**, 502 G 0.970*, 503 G 0.992**, 507 F 0.991**, 511 S 0.994**, 519 S 0.982*, 523 S 0.996**, 538 V 0.977* |
|  |  |  |  |  | f | 0.58855 | 0.10272 | 0.26286 | 0.04588 |  |  |  |
|  |  |  |  |  | ω0 | 0.07187 | 1.00000 | 0.07187 | 1.00000 |  |  |  |
|  |  |  |  |  | ω1 | 0.07187 | 1.00000 | 63.07884 | 63.07884 |  |  |  |
|  |  | Model A null | 71 | -42191.63 | 1 |  |  |  |  |  |  | Not Allowed |
|  | *nad6* | Model A | 72 | -11102.90 | Site class | 0 | 1 | 2a | 2b | Model A vs.Model A null | 0.0077 | 3 I 1.000**, 7 L 1.000**, 12 L 0.997**, 20 L 0.997**, 23 T 0.984*, 28 V 0.958*, 29 A 0.996**, 35 W 0.996**, 42 A 0.996**, 49 F 0.964*, 50 L 0.970*, 58 T 0.992**, 61 T 0.999**, 69 T 1.000**, 85 N 0.994**, 87 L 0.990*, 95 H 1.000**, 96 K 0.995**, 97 I 0.956*, 100 S 0.996**, 101 G 0.982*, 102 T 0.956*, 103 E 0.995**, 106 T 0.977*, 129 S 1.000**, 133 S 0.986* |
|  |  |  |  |  | f | 0.47135 | 0.07130 | 0.39725 | 0.06009 |  |  |  |
|  |  |  |  |  | ω0 | 0.06479 | 1.00000 | 0.06479 | 1.00000 |  |  |  |
|  |  |  |  |  | ω1 | 0.06479 | 1.00000 | 61.36780 | 61.36780 |  |  |  |
|  |  | Model A null | 71 | -11106.46 | 1 |  |  |  |  |  |  | Not Allowed |
|  | *cox1* | Model A | 72 | -24421.90 | Site class | 0 | 1 | 2a | 2b | Model A vs.Model A null | 0.0385 | 155 E 0.972* |
|  |  |  |  |  | f | 0.90038 | 0.02854 | 0.06889 | 0.00218 |  |  |  |
|  |  |  |  |  | ω0 | 0.01865 | 1.00000 | 0.01865 | 1.00000 |  |  |  |
|  |  |  |  |  | ω1 | 0.01865 | 1.00000 | 74.69725 | 74.69725 |  |  |  |
|  |  | Model A null | 71 | -24424.04 | 1 |  |  |  |  |  |  | Not Allowed |
|  | *cytb* | Model A | 72 | -23302.46 | Site class | 0 | 1 | 2a | 2b | Model A vs.Model A null | 0.0000 | 4 S 1.000**, 24 L 0.961*, 50 S 0.999**, 52 T 0.985*, 53 L 0.997**, 81 C 1.000**, 89 G 0.999**, 96 L 0.997**, 97 Q 1.000**, 99 H 0.999**, 111 T 0.997**, 147 D 0.997**, 150 K 0.958*, 179 A 0.999**, 181 A 0.966*, 197 L 0.998**, 237 L 0.998**, 244 T 0.984*, 250 T 0.979*, 253 I 0.999**, 273 N 0.984*, 279 L 0.968*, 282 L 0.993**, 283 L 0.998**, 294 T 0.998**, 298 K 0.999**, 307 N 1.000**, 324 L 0.967*, 335 F 0.997**, 356 T 0.998** |
|  |  |  |  |  | f | 0.00003 | 0.00000 | 0.96156 | 0.03841 |  |  |  |
|  |  |  |  |  | ω0 | 0.03404 | 1.00000 | 0.03404 | 1.00000 |  |  |  |
|  |  |  |  |  | ω1 | 0.03404 | 1.00000 | 1.00000 | 1.00000 |  |  |  |
|  |  | Model A null | 71 | -23269.10 | 1 |  |  |  |  |  |  | Not Allowed |
| *P. daviui* | *nad4* | Model A | 72 | -29345.91 | Site class | 0 | 1 | 2a | 2b | Model A vs.Model A null | 0.0134 | 23 S 0.951*, 37 S 0.970*, 48 S 0.974*, 60 A 0.989*, 84 L 0.983*, 260 V 0.971*, 284 S 0.996**, 319 W 0.959*, 379 E 0.973*, 390 G 0.990**, 411 S 0.958*, 413 I 0.956*, 414 F 0.989* |
|  |  |  |  |  | f | 0.78376 | 0.04107 | 0.16645 | 0.00872 |  |  |  |
|  |  |  |  |  | ω0 | 0.04528 | 1.00000 | 0.04528 | 1.00000 |  |  |  |
|  |  |  |  |  | ω1 | 0.04528 | 1.00000 | 13.69295 | 13.69295 |  |  |  |
|  |  | Model A null | 71 | -29348.97 | 1 |  |  |  |  |  |  | Not Allowed |
|  | *nad5* | Model A | 72 | -42222.23 | Site class | 0 | 1 | 2a | 2b | Model A vs.Model A null | 0.0362 | 6 A 0.970*, 20 S 0.955*, 23 V 0.982*, 132 Q 0.992**, 148 G 0.953*, 157 S 0.953*, 232 S 0.951*, 282 I 0.976*, 285 A 0.970*, 332 I 0.956*, 461 T 0.958*, 486 K 0.990* |
|  |  |  |  |  | f | 0.73648 | 0.11827 | 0.12515 | 0.02010 |  |  |  |
|  |  |  |  |  | ω0 | 0.07092 | 1.00000 | 0.07092 | 1.00000 |  |  |  |
|  |  |  |  |  | ω1 | 0.07092 | 1.00000 | 43.91410 | 43.91410 |  |  |  |
|  |  | Model A null | 71 | -42224.43 | 1 |  |  |  |  |  |  | Not Allowed |
| *B. jaraguensis* | *nad2* | Model A | 72 | -19779.55 | Site class | 0 | 1 | 2a | 2b | Model A vs.Model A null | 0.0001 | 162 M 0.959*, 233 F 0.973* |
|  |  |  |  |  | f | 0.79491 | 0.03611 | 0.16164 | 0.00734 |  |  |  |
|  |  |  |  |  | ω0 | 0.04900 | 1.00000 | 0.04900 | 1.00000 |  |  |  |
|  |  |  |  |  | ω1 | 0.04900 | 1.00000 | 1.28390 | 1.28390 |  |  |  |
|  |  | Model A null | 71 | -19771.49 | 1 |  |  |  |  |  |  | Not Allowed |
| *M. dominicanus* | *nad3* | Model A | 72 | -7323.42 | Site class | 0 | 1 | 2a | 2b | Model A vs.Model A null | 0.0190 | 81 N 0.995** |
|  |  |  |  |  | f | 0.85321 | 0.12507 | 0.01895 | 0.00278 |  |  |  |
|  |  |  |  |  | ω0 | 0.03891 | 1.00000 | 0.03891 | 1.00000 |  |  |  |
|  |  |  |  |  | ω1 | 0.03891 | 1.00000 | 161.98168 | 161.98168 |  |  |  |
|  |  | Model A null | 71 | -7326.17 | 1 |  |  |  |  |  |  | Not Allowed |
| *B. brachycaudus* | *atp6* | Model A | 72 | -15165.74 | Site class | 0 | 1 | 2a | 2b | Model A vs.Model A null | 0.0206 | 10 S 0.992**, 66 S 0.969*, 79 S 0.987*, 119 N 0.971*, 128 I 0.990**, 147 N 0.974*, 177 Y 0.982*, 184 G 0.990* |
|  |  |  |  |  | f | 0.70794 | 0.06884 | 0.20344 | 0.01978 |  |  |  |
|  |  |  |  |  | ω0 | 0.05148 | 1.00000 | 0.05148 | 1.00000 |  |  |  |
|  |  |  |  |  | ω1 | 0.05148 | 1.00000 | 44.53036 | 44.53036 |  |  |  |
|  |  | Model A null | 71 | -15168.42 | 1 |  |  |  |  |  |  | Not Allowed |
|  | *nad5* | Model A | 72 | -42197.28 | Site class | 0 | 1 | 2a | 2b | Model A vs.Model A null | 0.0001 | 1 S 0.977*, 12 G 0.959*, 13 S 0.991**, 45 S 0.980*, 73 S 0.972*, 74 S 0.953*, 97 G 0.956*, 130 S 0.972*, 132 Q 0.999**, 163 G 0.984*, 229 S 0.987*, 312 I 0.986*, 330 L 0.992**, 336 C 0.986*, 365 S 0.981*, 375 S 0.982*, 388 S 0.983*, 391 V 0.976*, 409 S 0.966*, 411 G 0.990**, 423 L 0.988*, 448 L 0.985*, 469 Y 0.983*, 472 G 0.991**, 479 W 0.991**, 485 Y 0.957*, 493 S 0.989*, 497 E 0.975*, 498 V 0.993**, 500 P 0.991**, 519 S 0.990* |
|  |  |  |  |  | f | 0.65345 | 0.10879 | 0.20383 | 0.03393 |  |  |  |
|  |  |  |  |  | ω0 | 0.07113 | 1.00000 | 0.07113 | 1.00000 |  |  |  |
|  |  |  |  |  | ω1 | 0.07113 | 1.00000 | 54.77953 | 54.77953 |  |  |  |
|  |  | Model A null | 71 | -42204.57 | 1 |  |  |  |  |  |  | Not Allowed |
| *S. allegheniensis* | *nad3* | Model A | 72 | -7325.19 | Site class | 0 | 1 | 2a | 2b | Model A vs.Model A null | 0.0375 | 77 V 0.990* |
|  |  |  |  |  | f | 0.85787 | 0.12736 | 0.01286 | 0.00191 |  |  |  |
|  |  |  |  |  | ω0 | 0.03868 | 1.00000 | 0.03868 | 1.00000 |  |  |  |
|  |  |  |  |  | ω1 | 0.03868 | 1.00000 | 108.89135 | 108.89135 |  |  |  |
|  |  | Model A null | 71 | -7327.36 | 1 |  |  |  |  |  |  | Not Allowed |
| *S. indentatus* | *atp8* | Model A | 72 | -4020.49 | Site class | 0 | 1 | 2a | 2b | Model A vs.Model A null | 0.0028 | 12 F 0.994**, 14 I 0.981* |
|  |  |  |  |  | f | 0.30650 | 0.45936 | 0.09370 | 0.14043 |  |  |  |
|  |  |  |  |  | ω0 | 0.09877 | 1.00000 | 0.09877 | 1.00000 |  |  |  |
|  |  |  |  |  | ω1 | 0.09877 | 1.00000 | 999.00000 | 999.00000 |  |  |  |
|  |  | Model A null | 71 | -4024.97 | 1 |  |  |  |  |  |  | Not Allowed |
|  | *nad5* | Model A | 72 | -42219.00 | Site class | 0 | 1 | 2a | 2b | Model A vs.Model A null | 0.0001 | 3 S 0.974*, 59 S 0.991**, 171 S 0.967*, 310 S 0.989*, 321 V 0.970*, 419 L 0.982*, 468 A 0.985*, 469 Y 0.968*, 486 K 0.986* |
|  |  |  |  |  | f | 0.79435 | 0.13501 | 0.06038 | 0.01026 |  |  |  |
|  |  |  |  |  | ω0 | 0.07100 | 1.00000 | 0.07100 | 1.00000 |  |  |  |
|  |  |  |  |  | ω1 | 0.07100 | 1.00000 | 13.75919 | 13.75919 |  |  |  |
|  |  | Model A null | 71 | -42227.16 | 1 |  |  |  |  |  |  | Not Allowed |
|  | *cox3* | Model A | 72 | -16038.31 | Site class | 0 | 1 | 2a | 2b | Model A vs.Model A null | 0.0150 | 118 T 0.989* |
|  |  |  |  |  | f | 0.92090 | 0.06266 | 0.01539 | 0.00105 |  |  |  |
|  |  |  |  |  | ω0 | 0.03997 | 1.00000 | 0.03997 | 1.00000 |  |  |  |
|  |  |  |  |  | ω1 | 0.03997 | 1.00000 | 29.92053 | 29.92053 |  |  |  |
|  |  | Model A null | 71 | -16041.27 | 1 |  |  |  |  |  |  | Not Allowed |
